# DNA methylation and gene expression regulation associated with vascularization in *Sorghum bicolor*

**DOI:** 10.1111/nph.14448

**Published:** 2017-02-10

**Authors:** Gina M. Turco, Kaisa Kajala, Govindarajan Kunde‐Ramamoorthy, Chew‐Yee Ngan, Andrew Olson, Shweta Deshphande, Denis Tolkunov, Barbara Waring, Scott Stelpflug, Patricia Klein, Jeremy Schmutz, Shawn Kaeppler, Doreen Ware, Chia‐Lin Wei, J. Peter Etchells, Siobhan M. Brady

**Affiliations:** ^1^ Department of Plant Biology and Genome Center UC Davis Davis CA 95616 USA; ^2^ DOE Joint Genome Institute 2800 Mitchell Drive Walnut Creek CA 94598 USA; ^3^ Cold Spring Harbor Laboratory 1 Bungtown Road Cold Spring Harbor NY 11724 USA; ^4^ Department of Agronomy and Great Lakes Bioenergy Research Center University of Wisconsin 1575 Linden Drive Madison WI 53706 USA; ^5^ Institute for Plant Genomics and Biotechnology and Department of Horticultural Sciences Texas A and M University College Station TX 77843 USA; ^6^ HudsonAlpha Institute for Biotechnology 601 Genome Way NW Huntsville AL 35806 USA; ^7^ USDA‐ARS Ithaca NY 14853 USA; ^8^ School of Biological and Biomedical Sciences Durham University South Road Durham DH3 1LE UK

**Keywords:** biofuel, cell type‐specific, epigenetics, sorghum (*Sorghum bicolor*), transcriptome

## Abstract

- Plant secondary cell walls constitute the majority of plant biomass. They are predominantly found in xylem cells, which are derived from vascular initials during vascularization. Little is known about these processes in grass species despite their emerging importance as biomass feedstocks. The targeted biofuel crop *Sorghum bicolor* has a sequenced and well‐annotated genome, making it an ideal monocot model for addressing vascularization and biomass deposition.
- Here we generated tissue‐specific transcriptome and DNA methylome data from sorghum shoots, roots and developing root vascular and nonvascular tissues.
- Many genes associated with vascular development in other species show enriched expression in developing vasculature. However, several transcription factor families varied in vascular expression in sorghum compared with Arabidopsis and maize. Furthermore, differential expression of genes associated with DNA methylation were identified between vascular and nonvascular tissues, implying that changes in DNA methylation are a feature of sorghum root vascularization, which we confirmed using tissue‐specific DNA methylome data. Roots treated with a DNA methylation inhibitor also showed a significant decrease in root length.
- Tissues and organs can be discriminated based on their genomic methylation patterns and methylation context. Consequently, tissue‐specific changes in DNA methylation are part of the normal developmental process.

## Introduction

Secondary cell walls (SCWs) such as those in the xylem constitute the majority of plant biomass and are an abundant source of biomaterials. It has been suggested that the sugars in SCWs could represent a renewable source of energy (Farrell *et al*., 2006; Somerville, 2006). Vascularization, or development of functional conductive tissue from undifferentiated cells, is relatively well understood in the dicot *Arabidopsis thaliana*, particularly for xylem tissue (see Růžička *et al*. (2015), and Kumar *et al*. (2016) for recent reviews). By contrast, little is known about vascular specification and xylem differentiation in monocots despite their emerging importance as biomass feedstock for the emerging bioenergy industry (Somerville, 2006). *Sorghum bicolor* has a sequenced, well‐annotated genome (Paterson *et al*., 2009) and is a biomass crop (Rooney *et al*., 2007; Mullet *et al*., 2014), making it an ideal model for addressing xylem development in monocots. Monocot and dicot SCWs differ in a number of ways. Dicots contain walls comprising cellulose and xyloglucan in roughly equal amounts, present in a pectin‐abundant gel. In commelinoid monocot walls, glucoarabinoxylans are the major hemicelluloses as opposed to xyloglucan, while cell walls are pectin‐poor (Carpita, 1996). The SCWs of monocots also contain higher amounts of lignin and, in some cases, silica (Vogel, 2008).

Here we ask which genes regulate vascular development in the monocot Sorghum relative to the well‐studied species, *Arabidopsis thaliana*, by generating tissue‐specific transcriptome data from developing vascular and nonvascular tissue in sorghum roots. We compared sorghum vascular‐expressed genes with those from Arabidopsis and maize to determine similarities and differences (Brady *et al*., 2007a; Stelpflug *et al*., 2015). Many sorghum genes with enriched expression in developing vasculature were orthologous to genes associated with vascular development in other species. However, several transcription factor families were overrepresented in sorghum compared with Arabidopsis, suggesting differential wiring of vascular regulatory networks. Surprisingly, gene ontology (GO) categories associated with DNA methylation were among those enriched in vascular cell types. Differentially methylated regions (DMRs) were subsequently identified between vascular tissue and nonvascular tissue, providing functional analysis to support our transcriptomic data. Our results provide a framework for understanding genetic and epigenetic changes linked to vascularization and SCW development in sorghum.

## Materials and Methods

### Access to data

Sorghum RNA‐seq and sodium bisulfite sequencing (SBS‐seq) were deposited in NCBI GEO under the accession GSE70903. Maize RNA‐seq data are available through NCBI accession numbers PRJNA171684 and SRP010680 (Stelpflug *et al*., 2015). Data analysis pipelines are publicly available (https://github.com/gturco/bs_seq_analysis).

### Nucleic acid preparation

*Sorghum bicolor* (L.) Moench (BTx623) seeds were grown in three biological replicates under sterile conditions, maintained in long days. Whole‐root/shoot RNA was isolated using TRIzol reagent (Life Technologies Corp., Carlsbad, CA, USA), followed by DNase treatment using RQ1 enzyme (Promega, Madison, WI, USA). Whole root/shoot methylomes were prepared from DNA extracted as previously described (Shure *et al*., 1983). Root vascular tissue was prepared for laser capture microdissection (LCM) as previously described (Kerk *et al*., 2003). Eight‐ to 10‐micron sections were mounted on PEN membrane slides (Leica Biosystems Inc., Buffalo Grove, IL, USA). Tissue types were separated using the LCM setup previously described (Belmonte *et al*., 2013). RNA extraction on LCM captures was performed using an RNAqueous‐Micro Kit (Ambion, Foster City, CA, USA) while a QIAamp Micro DNA kit (Qiagen) was used for DNA extraction.

### RNA‐seq

For whole‐root/shoot RNA‐seq, mRNA was purified from 1 μg of total RNA using magnetic beads containing poly‐T oligos. LCM RNA‐seq libraries were prepared by removing rRNA from 10 to 50 ng of total RNA using Ribo‐Zero rRNA Removal Kit (Epicentre, Madison, WI, USA). Stranded cDNA libraries were generated using the Truseq Stranded RNA LT kit (Illumina, San Diego, CA, USA). mRNA was fragmented using divalent cations and high temperature. Fragmented RNA was reverse‐transcribed using random hexamer primers and SSII (Invitrogen) followed by second strand synthesis. Fragmented cDNA was treated with end‐repair, A‐tailing, adapter ligation, and 10 cycles of PCR (for whole root/shoot) or 15 cycles of PCR (for LCM captured material). Quantitative PCR (qPCR) was used to determine library concentration. Libraries of three biological replicates for each tissue/organ were sequenced on Illumina Hiseq.

RNA‐seq data were filtered for poor quality and adapter sequences then aligned with TopHat (Trapnell *et al*., 2009) using the Phytozome *Sorghum bicolor 2.0* (BTx623) genome as the reference under default parameters. Differential expression was determined with edgeR (McCarthy *et al*., 2012), which normalized data and ran a genewise exact test. Fragments per kilobase of transcript per million mapped read values among biological replicates were highly correlated with *r* ^2^ values of 0.98–0.94 (see later Supporting Information Figs S2, S3). Genes with a false discovery rate (FDR) ≤ 0.05 and a positive log fold‐change were considered differentially expressed. Genes with at least three counts per million (CPM) across all three replicates were considered expressed. Reads per kilobase per million (RPKM) were categorized into four groups based on quartile range where 1 delineates the lowest and 4 the highest expression quartile.

### qRT‐PCR for RNAseq validation

Two independent biological replicates of LCM‐isolated vascular and nonvascular samples were used for quantitative reverse transcription polymerase chain reaction (qRT‐PCR). First‐strand cDNA was synthesized with SuperScriptIII reverse transcriptase (Invitrogen) and oligo(dT) primers. qRT‐PCR was carried out using iCycler iQ Real‐Time PCR Detection System (Bio‐Rad, Hercules, CA, USA) using the Bio‐Rad iQ SYBR green Supermix. Primers used are listed in Table S1 The comparative 2^ΔΔ*C*T^ method was used to quantify relative abundance of transcripts (Livak & Schmittgen, 2001) in vascular vs nonvascular tissues. *ACTIN11* (*Sb01g010030*) was chosen as a reference gene based on previous expression data.

### GO enrichment and cross‐species comparisons

The python program goatools (https://github.com/gturco/goatools) was used to determine GO enrichment. Bonferroni‐corrected ontologies with *P*‐values < 0.05 were considered enriched. GO consortium files in gene association format were obtained from Gramene (http://www.gramene.org/).

For comparison of vascular and nonvascular expressed sorghum genes with Arabidopsis orthologs, data from Brady *et al*. (2007a), vascular tissue marker lines WOL, J2501, J0121, APL, S17, S32, S4, SUC were compared with genes expressed in nonvascular marker lines COBL9, CORTEX, GL2, J0571, PET111, LRC, SCR5 and AGL42. To be considered vascular‐enriched, transcript abundance in one vascular cell type was > 1.2‐fold higher than that in all nonvascular cell types, at a significance level of *q* ≤ 0.001. Arabidopsis genes were considered vascular‐expressed if they had an expression value > 1 in any vascular marker line described earlier. Maize data were obtained from Stelpflug *et al*. (2015). Cortical parenchyma (epidermis, cortex, endodermis) and stele tissue samples from the root of 3‐d‐old seedlings were used for comparisons. Differentially expressed genes and vascular‐expressed genes were determined in edgeR as described earlier.

Transcription factors were annotated using the Grassius TFome collection. A Fisher exact test was used to test for vascular enrichment among species where the number of vascular‐expressed transcription factors for each family over the total number of transcription factors for the family was compared with the number of vascular‐expressed genes in that species over the total number of genes in the genome.

### Coexpression

The reference gene for each analysis was compared with the RPKM values of each tissue type – root, shoot, root vascular, root nonvascular and embryo – for all genes with expression profiles across all tissue types. The Pearson correlation was taken for all combinations of reference gene lines (RPKM of one tissue type connecting RPKM of a second tissue type) compared with all combinations of potential RPKM lines for the query gene. Correlations ≥ 0.9 were used for GO enrichment analysis.

### 5‐Azacytidine treatment

Surface‐sterilized seeds were grown at 22**°**C, 74% humidity using a 12 h light cycle on Whatman paper in a Petri dish that contained 20 ml supplemented with 0.05% silwet and either 5‐azacytidine solution or dimethyl sulfoxide  control. A dose–response gradient of methyltransferase inhibitor at 20, 50 and 100 μM 5‐azacytidine treatment was performed. At the 100 μM concentration at 4 d postgermination (DPG), roots appeared shorter but had no signs of phototoxicity in shoots, consistent with previous reports suggesting that 100 μM 5‐azacytidine is nontoxic in sorghum (Emani *et al*., 2002). For measurements, 6 μl of 1 mM 5‐azacytidine‐stock solution were added to 54 μl water for growing seedlings. These were compared with seedling grown in 54 μl water supplemented with 6 μl of the DMSO control. Samples (*n *=* *60) were collected from random positions of the three biological replicate plates. Randomly selected roots were scanned and their length quantified with the imageJ plug in smartroot (Lobet *et al*., 2011). To test for significance a two‐way ANOVA to root length ~ replicate × treatment was used.

### DNA methyl‐Seq

Whole‐root and whole‐shoot methylomes were generated from 1 μg of DNA which was sheared to 500 bp using the Covaris LE220 (Covaris Inc., Woburn, MA, USA). DNA fragments smaller than 200 bp were removed using SPRI beads (Beckman Coulter, Brae, CA, USA). Remaining fragments were treated for end‐repair, A‐tailing, and ligation of methylated Illumina adapters using an Illumina library creation kit (KAPA Biosystems, Wilmington, MA, USA). Adaptor‐ligated DNA was bisulfite treated using the EZ DNA Methylation Lightning Kit (Zymo Research Corp., Irvine, CA, USA). Converted DNA was enriched with 10 cycles of PCR. qPCR was used to determine the concentration of the libraries. Libraries of three biological replicates for each tissue type were sequenced on an Illumina Hiseq. Vascular and nonvascular root methylomes were generated similarly, but from 100 ng of DNA, which was sheared to 700 bp. Enrichment was with 12 cycles of PCR to generate the final library.

Filtered high‐quality sequences were mapped using bsmap with default parameters (Xi & Li, 2009). The Smithlab DNA Methylation Data Analysis Pipeline (Song *et al*., 2013) was used for identification of CpG, CHG and CHH methylated regions and removal of regions of PCR overamplication. Methylation counts were merged for each of the three biological replicates using merge‐methcounts (Song *et al*., 2013). Spikes in controls were used to determine error rates (FDR < 0.01). A binomial test was applied to each cytosine with at least 4× read depth (level of coverage used in Zhong *et al*., 2013b), calculated as *B*(*x *≥ *k*;* n*,* p*) for each cytosine, where *n* represents read depth, *k* the number of methylated cytosines, and *p* the expected rate of error obtained from spike‐in controls (0.01–0.33%). Methylated cytosines were identified after correcting for multiple testing at an FDR ≤ 0.01.

To evaluate the similarity of methylation between biological replicates and across tissue types, we used principal component analysis (PCA) to reduce the dimensionality of our data and to explore variation between each tissue/organ dataset. Sites with BS‐sequencing coverage ≥ 4× were used for analysis, were included if they had sufficient coverage in each biological replicate in all tissues/organ types and were determined as methylated or unmethylated using the binomial test described earlier. Independent validation of similarity between biological replicates was performed using a Pearson correlation of methylation status of sites shared between tissues/organs.

Methylation averages across genic regions were calculated using a sliding‐window approach. A window size of 20 bp was used for regions 2000 bp directly upstream and downstream of coding regions. Gene‐body methylation was determined using exons concatenated into bins of 100 (Regulski *et al*., 2013). The number of methylated marks in each window/bin was compared with the total number of cytosines with ≥ 4× coverage for that bin. The methylation average within a bin was summed and averaged across all genes of that region. A nonparametric ANOVA determined if differences seen in average methylation between groups (varying levels of RPKM and/or between tissue types) was significant. We performed a Kruskal–Wallis test followed by a Tukey test for pairwise comparisons on average methylation within the 50 genomic bins upstream of genes, 100 genomic bins (representing the gene body) and 50 genomic bins downstream of genes, for each group (varying levels of RPKM and/or tissue type comparisons).

### Identification of DMRs

A sliding window of 100 bp bins at 50 bp iterations was used to identify DMRs. All three DNA methylation contexts were used to determine differential methylation. Cytosines with ≥ 4× coverage in both comparisons were used. A Fisher exact test was run on each window that contained at least 15 cytosines (Stroud *et al*., 2013; Zhong *et al*., 2013b). Adjacent windows with a *P*‐value < 0.001 were concatenated. Regions with a methylation < 30% in one sample and > 70% in the other were referred to as DMRs. Genes were considered overlapping with DMRs if they intersect 1000 bp 5′ and 1000 bp 3′ of the gene. To call hypermethylated regions in a single tissue type relative to the background, a one‐tailed binomial test was run on each gene, and the total number of cytosines that were at least 4× coverage for each context was used as parameter *N* (only genes with 20 or more cytosines were used), the number of methylated cytosines was used as parameter *K* and the average genome‐wide methylation was used as *P*. A Benjamini–Hochberg FDR was used, *P*‐values ≤ 0.05 were considered significant (Kawakatsu *et al*., 2016). K‐means clustering was used to cluster DMRs of each tissue type.

## Results

### Vascular tissue transcriptome in sorghum roots

**Figure 1:**
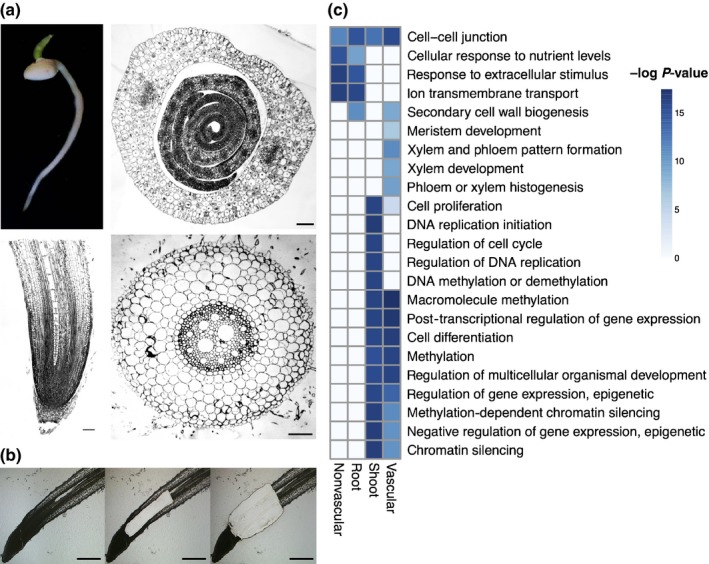
Transcriptional profiling of sorghum tissues. (a) Upper panel, 3‐d‐old sorghum root and shoot tissues and respective shoot cross0section. Bar, 100 µm. Lower panel, whole sorghum root and root cross section with 400 µm scale. (b) Laser capture microdissection (LCM) capturing the root vascular tissue from meristematic zone through the elongation zone. The left panel shows the whole root, the middle panel has the vascular section captured, and the right panel has both the vascular and nonvascular sections captured. (c) Selected gene ontology (GO) terms enriched for differentially expressed genes up‐regulated between root and shoot, and between root vascular and nonvascular tissues. A darker color blue indicates a more significant *P*‐value. All GO terms have a *P*‐value < 0.005 in at least one of the tissue types.

Genes required for xylem cell proliferation and their subsequent differentiation are expressed in the vascular cylinder of the root meristem and elongation zone, before the deposition of the secondary cell walls in the maturation zone (Brady *et al*., 2007a), making the root tip an excellent system for analyzing xylem development. To determine transcriptomic differences of vascular cell types in comparison to that of other cells, we used LCM to separate vascular and nonvascular cell types from the meristem and elongation zone of 3‐d‐old *Sorghum bicolor* BTx623 roots (Fig. 1a). Whole shoots and roots from 3‐d‐old plants were also collected for comparison. Vascular cells were collected with cuts below the first fully differentiated xylem vessel and above the quiescent center along the radial axis of the root, and along the pericycle cell file, parallel to the longitudinal axis (Fig. 1a,b). Nonvascular cells were isolated from the remaining tissue and from the same position along the root longitudinal axis. Three biological replicates were collected for each of these four samples, which were subjected to RNA‐seq from paired‐end reads of 150 bp in size. Biological replicates between samples were highly correlated with an *r* ^2^ from 0.92 to 0.95 (Figs S1, S2). Individual replicates from whole‐root and whole‐shoot samples contained a depth of coverage of 60–90 million mapped reads, and 41–46 million mapped reads for vascular and nonvascular tissues (Table S2, S3). Differentially expressed genes were identified between vascular and nonvascular tissues, and between root and shoot organs with edgeR using an FDR ≤ 0.05 (Tables [Link], [Link]). To ensure successful isolation of LCM tissues and characterization of differential expression, qRT‐PCR was performed on tissue‐specific marker genes (Fig. S3). Expression differences elucidated by qRT‐PCR matched the differential expression patterns using RNA‐seq and edgeR (Fig. S3), thus verifying our dataset.

Between shoot and root organs, 8237 differentially expressed genes were identified; 5022 differentially expressed genes were identified between root vascular and nonvascular tissue. Of these, 2291 genes demonstrated higher vascular expression and 2731 genes were preferentially expressed in nonvascular tissue (Table S5). To gain an overview of underlying biological programmes that differed between samples, a GO enrichment analysis was performed on differentially expressed genes (Fig. 1c; Table S6). Genes enriched in the whole‐root and nonvascular tissue relative to shoot and vascular tissue demonstrated overrepresentation of categories, including cellular response to nutrient concentrations (Bonferroni corrected *P *=* *1.37e–6), ion membrane transport (*P *=* *4.17e–7) and response to extracellular signals (*P *=* *4.14e–7), consistent with the role of the root in the uptake of water and nutrients. Whole‐root and vascular tissue transcriptomes demonstrated enrichment in expression of genes associated with SCW biogenesis (*P* = 5.07e–5 and 0.0003, respectively). mRNA from vascular‐enriched tissue was enriched for xylem and phloem pattern formation (*P *=* *4.32e–5), phloem and xylem histogenesis (*P *=* *0.000 158) and xylem development (*P *=* *0.000 261), suggesting that we have successfully captured the transcriptome of the developing vasculature of the root.

### Comparisons between vascular‐expressed genes in monocots and dicots

**Figure 2:**
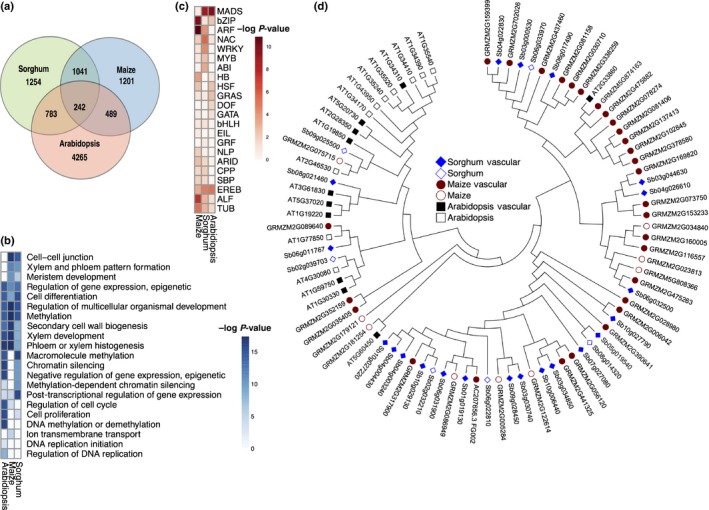
Conservation and divergence of vascular‐expressed genes in monocots and dicots. (a) Number of vascular‐enriched genes shared and unique to sorghum, maize and Arabidopsis. (b) Selected gene ontology (GO) term enrichment for vascular‐expressed genes between species; darker blue corresponds to a lower *P*‐value. (c) Expansion and conservation of annotated transcription factor families expressed in vascular tissue. Enrichment of a family in vascular tissue is indicated by a darker color red. A Fisher exact test was used to test for vascular enrichment among species where the number of vascular‐expressed transcription factors for each family over the total number of transcription factors for the family was compared with the number of vascular‐expressed genes in that species over the total number of genes in the genome. (d) Auxin response factor (ARF) transcription factor family phylogeny across sorghum, maize and Arabidopsis. Filled symbols represent vascular‐enriched genes within the ARF family. Sorghum and maize have an expansion of ARFs in comparison to Arabidopsis.

We reasoned that our vascular‐enriched transcript data could be used to identify potential similarities and differences between control of vascular development in monocots and dicots where comparable data were available. The *WOODEN LEG* (*WOL*) gene of Arabidopsis is expressed specifically in the root vasculature with enrichment in the meristem (Mähönen *et al*., 2000). Arabidopsis *pWOL*::*GFP* expressing cells have previously been isolated and used for transcriptomic analysis (Brady *et al*., 2007a), providing comparable data to that generated for sorghum root vascularization. A comparable tissue‐specific RNA‐seq dataset from maize comprising vascular and nonvascular cell types from a similar developmental point along the root on the apical‐basal axis as that derived from sorghum was also used for comparison (Stelpflug *et al*., 2015). We identified 242 homologs between vascular‐enriched transcripts of all three species (Fig. 2a; Table S7). Vascular‐enriched genes, shared by sorghum, maize and Arabidopsis roots (Fig. 2b; Table S7), demonstrated enrichment for xylem development (*P* = 4.31e–5) and SCW biogenesis (*P* = 0.0003).

Many transcription factors associated with control of vascular development and SCW differentiation have been characterized (Brown *et al*., 2005; Kubo *et al*., 2005; Zhong *et al*., 2006, 2008, 2011, 2013a; Mitsuda *et al*., 2007; Zhou *et al*., 2009; Zhong & Ye, 2012; Taylor‐Teeples *et al*., 2015). To assess whether the number of genes encoding these family members had undergone differential expansion in sorghum vascular tissue (Figs 2c, S4), we compared our data with those of maize and Arabidopsis. Changes to the complement of each transcription factor family that demonstrated expression in vascular tissue could point to subtle differences in regulatory networks between species. A Fisher exact test, demonstrated that NAC and MYB transcription factor family genes were both overrepresented in sorghum vasculature (*P* = 3.16e–6 and 0.002, respectively) compared with maize and Arabidopsis (Fig. 2c). WRKY, CPP and ABI transcription factors families were also overrepresented in sorghum compared with the other species tested (Fig. 2c). Auxin response factor (ARF) and ABI3‐like factor (ALF) (Fig. 2c,d, S4) families demonstrated the largest divergence in vascular regulation between monocots and dicots. The ARF family demonstrated the largest divergence in vascular‐expressed genes between monocots and dicots, containing over double the number of vascular‐expressed transcription factors belonging to this family (21 vascular expressed transcription factors in sorghum, 29 in maize and 11 in Arabidopsis). The left‐hand bottom branch of the ARF family phylogeny (Figs S4A, 2d) shows only monocot genes, most of which are vascular‐expressed. This suggests differential wiring of the underlying transcriptional network associated with vascular development among these species.

### An association between DNA methylation and vascularization

**Figure 3:**
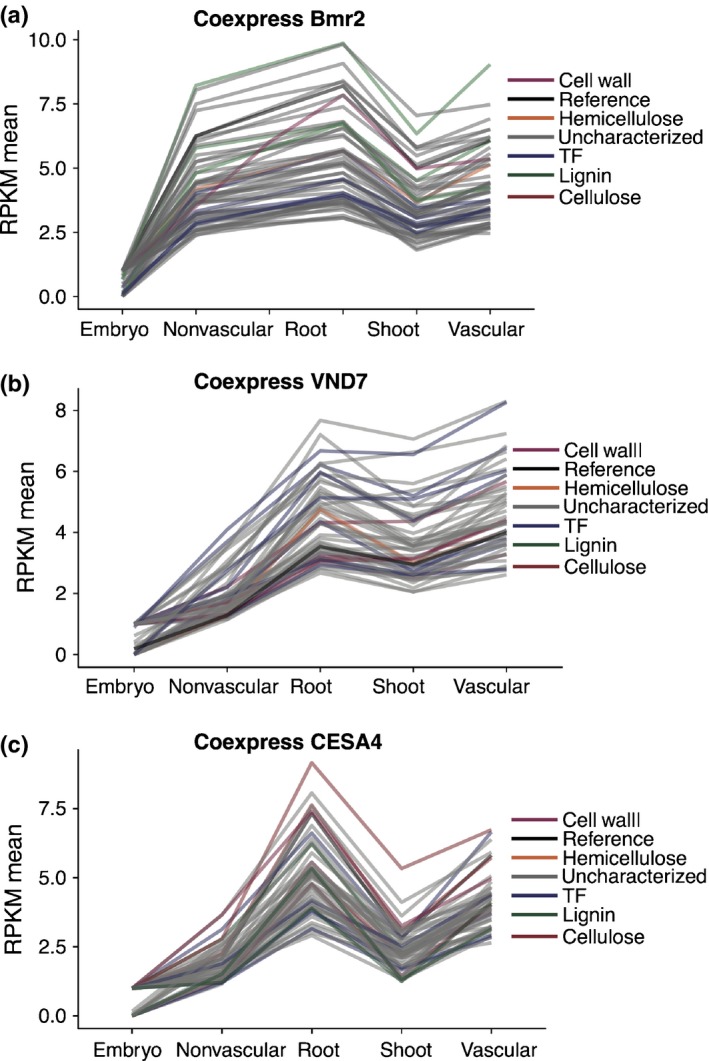
Identification of genes associated with vascular development in sorghum through coexpression analysis. Top 50 genes coexpressed with *Bmr2*,*VND7* and *CESA4* (a, b and c, respectively). Coexpression was determined by correlating the slope of the line for each gene's reads per kilobase per million (RPKM) expression value across tissues against the reference gene's slope. Genes with a Pearson correlation ≥ 0.9 were considered coexpressed. Sorghum genes with known functions in secondary cell wall biosynthesis have been colored according to function. TF, transcription factor.

In Arabidopsis*,* the expression of many genes associated with SCW biosynthesis is coregulated. Consequently, identification of coexpressed genes was used as a tool to identify additional genes associated with this process (Brown *et al*., 2005; Persson *et al*., 2005; Zhong *et al*., 2008). Identifying orthologous genes in the Poaceae that are associated with SCW synthesis is not straightforward, as these species diverged *c*. 200 million yr ago. There are large expansions and reductions in gene content within several transcription factor families associated with SCW synthesis (Fig. 2b), and for other SCW genes such as members of the COBRA family (Brady *et al*., 2007b) that are required for cellulose deposition (Li *et al*., 2003). We used our gene expression profiles to further annotate SCW biosynthetic or regulatory genes in sorghum using coexpression analyses. In addition to our data, we also included a previously described dataset from sorghum embryos (Olson *et al*., 2014). As ‘bait’ we used the ortholog of maize *Brown midrib 2* (*Bmr2*), required for SCW lignification (Saballos *et al*., 2012); an Arabidopsis SCW cellulose synthase ortholog (*CESA4*/*IRX5*) (Taylor *et al*., 2003); and *VND7*, a xylem master regulator (Kubo *et al*., 2005) expressolog (homolog with similar expression pattern) (Patel *et al*., 2012; Fig. 3b; Table S8). Many genes with high coexpression profiles had previously been annotated as SCW‐associated, but many genes not previously linked to SCW biosynthesis were also identified (Tables 1, S8; Fig. 3). GO analysis was carried out on coexpressed genes (Pearson coefficient ≥ 0.9; Table S8). In common with analyses carried out using our differentially expressed gene data, enrichment of the GO category xylem and phloem pattern formation was present in genes coexpressed with *VND7* (*P *=* *1.50e‐4). New terms were also identified, including genes associated with the mediator complex (*P *=* *4.21e–3), which was linked with the growth response to reductions in lignin deposition in the secondary cell wall (Bonawitz *et al*., 2014). In order to identify genes that are likely to be involved in secondary cell wall biosynthesis in xylem cells, we next identified genes that are correlated with *CESA4, VND7* or *Bmr2* with a Pearson correlation threshold > 0.9 and which are also present in the vasculature‐enriched gene set. This set of high‐confidence xylem cell secondary cell wall biosynthesis genes are further described in Table S8 and Fig. S5

**Table 1:** Expression correlation with cell wall associated genes

Pearson coefficient	Gene ID	Description	Function
Correlated with IRX5/CESA4 (cellulose synthase; Sb01g019720)			
0.999	Sb02g025020	IRX3; cellulose synthase	Cellulose
0.998	Sb02g038740	IRX6/COBL4	Cellulose
0.953	Sb03g034680	IRX1; cellulose synthase	Cellulose
0.982	Sb10g005780	HCT; Hydroycinnamoyl‐COA shikamate	Lignin
0.973	Sb03g010240	Peroxidase 72 (PER72) (P72) (PRXR8)	Lignin
0.972	Sb09g022460	LAC17; laccase 17	Lignin
0.997	Sb03g034930	C3HC4‐type RING finger family protein	TF
0.986	Sb07g001550	Embryo Defective 2301	TF
0.975	Sb03g032260	MYB86 transcriptional repressor	TF
0.959	Sb06g027620	C3HC4‐type RING finger family	TF
0.954	Sb03g002660	ATHB‐15; HD‐Zip III	TF
0.991	Sb06g032310	LRR family/extensin family	Cell wall protein
0.980	Sb01g038450	ESK1 (ESKIMO 1)/DUF231	Cell wall‐related
0.970	Sb06g015880	TCH4; xyloglucan:xyloglucosyl transferase	Cell wall‐modifying
Correlated with *BMR2*; 4‐coumarate‐CoA ligase (4CL1; Sb04g005210)			
0.977	Sb01g038640	IRX9; xylosyltransferase	Hemicellulose
0.993	Sb06g030260	Putative shikimate kinase	Lignin
0.977	Sb01g048200	OPCL1; 4‐coumarate‐CoA ligase	Lignin
0.976	Sb04g026510	PAL2; phenylalanine ammonia‐lyase	Lignin
0.989	Sb05g003240	bZIP family transcription factor	TF
0.989	Sb05g002940	DUF296; DNA‐binding family protein	TF
0.985	Sb04g035890	Basic helix–loop–helix (bHLH) family	TF
0.983	Sb10g023210	IAA1; protein binding/transcription factor	TF
0.977	Sb09g004315	Zinc finger (GATA type) family protein	TF
0.992	Sb07g025170	ATBAG1 (BCL2‐associated athanogene 1)	PCD
NAC007/VND7 transcription factor (Sb10g000460)			
0.977	Sb03g006950	RXF12; endo‐1,4‐beta‐xylanase/hydrolase	Hemicellulose
0.994	Sb03g000840	Basic helix–loop–helix (bHLH) family	TF
0.989	Sb02g030180	ZPR1 (LITTLE ZIPPER 1); protein binding	TF
0.989	Sb04g010670	Basic helix–loop–helix (bHLH) family	TF
0.986	Sb01g033470	Basic helix–loop–helix (bHLH) family	TF
0.985	Sb05g003050	Zinc finger (C3HC4‐type RING finger) family	TF
0.981	Sb03g011680	IAA31; protein binding/transcription factor	TF
0.977	Sb09g007650	Basic helix–loop–helix (bHLH) family	TF
0.977	Sb06g025650	LHW (bHLH)	TF
0.991	Sb02g024050	AtGH9B8 (glycosyl hydrolase 9B8)	Cell wall‐organizing
0.991	Sb04g034400	Peptidoglycan‐binding LysM domain‐containing	Cell wall‐modifying
0.989	Sb04g032820	AtEXPB4 (expansin)	Cell wall‐organizing
0.987	Sb01g009770	Cation binding/*O*‐glycosyl hydrolase	Cell wall‐organizing

Genes were categorized as either lignin, cellulose, hemicellulose, programmed cell death (PCD), transcription factor (TF) or other cell wall‐related functions.

During our transcriptome analyses we also observed a number of unexpected enriched GO categories. Regulation of gene expression, both epigenetic (*P *=* *3.56e–6) and methylation (*P *=* *4.85e–7), was enriched in vascular tissue compared with nonvascular tissue (Table S6). Strikingly, these GO categories were also enriched in *Bmr2* and *VND7* coexpression groups (*P *=* *4.93e–7 and 1.38e–6, respectively; Table S8). Furthermore, the DNA methylation GO category was also enriched with genes coexpressed with *Bmr2* (*P* = 1.14e–5; Table S8). Genes enriched in vascular tissue associated with methylation included a homolog of *AGO4* that links 24 nt small interfering RNA (siRNA) binding and RNA‐directed DNA methylation and that potentially triggers *de novo* DNA methylation (Zilberman *et al*., 2003; Ye *et al*., 2016); and all three sorghum homologs of *DCL2* and two of the four *DCL4* homologs – *DCL2* and *DCL4*, in conjunction with *DCL3*, act in a partially redundant fashion to regulate nonCG DNA methylation (Henderson *et al*., 2006). In addition, the genes SUVH4 and SUVH5 regulate maintenance of CHG methylation and related H3K9me2 (Ebbs & Bender, 2006; Vogel, 2008; Rajakumara *et al*., 2011).

In plants, DNA methylation, present in three contexts, CG, CHG, and CHH, is dynamic during development. Small RNAs (sRNAs) contribute to DNA methylation through RNA‐directed DNA methylation and gene silencing. Collectively, these sRNAs silence gene expression through parallel post‐transcriptional and translational silencing and DNA methylation. A small number of examples of methylation regulating expression of single genes during development are described in the literature (Gutierrez‐Marcos *et al*., 2006; Jiang *et al*., 2007; Tolley *et al*., 2012; Rodrigues *et al*., 2013; Zhong *et al*., 2013b). Consequently, we hypothesized that DNA methylation may differ in vascular and nonvascular contexts in the root.

**Figure 4:**
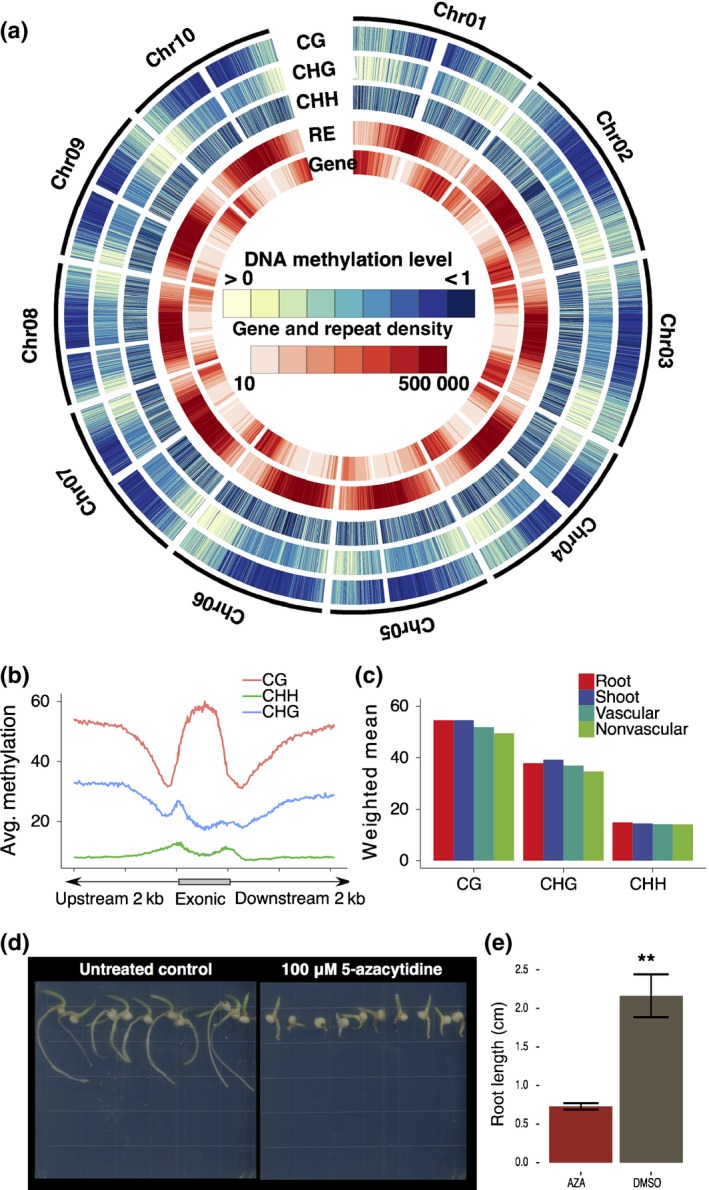
Sorghum methylome and genome architecture. Sodium bisulfite sequencing data across three biological replicates were merged and used in calling methylated regions for each tissue type. (a) Sorghum epigenome density plot for CG, CHG and CHH methylation contexts in vascular tissue. A darker color blue corresponds to a higher percentage methylation within a 10 000 bp window. Repetitive elements (RE) and gene density are reported on the inner tracks, where a darker color red corresponds to a higher density. (b) DNA methylation patterns averaged across genic regions for each methylation context in vascular tissue. Vascular methylation patterns shown here are representative of all tissue types. (c) Weighted means of genome‐wide average methylation in each methylation context and each sample. (d) Sorghum seedlings grown on DNA methylation inhibitor 5‐azacytidine. Ten randomly selected plants 4 d postgermination (DPG) grown on either 100 μM 5‐azacytidine dissolved in dimethyl sulfoxide (DMSO) or on DMSO (untreated). (e) Quantification of root lengths of sorghum seedlings grown on either 100 μM 5‐azacytidine (AZA) or DMSO. (e) **, *P *<* *0.005 for replicate × treatment interaction term determined using a two‐way ANOVA fit to root length ~ treatment × replicate. The error bars represent ± SE between samples (*n *=* *30).

To determine whether DNA methylation influences sorghum root development, seedlings were subjected to a nontoxic concentration (100 μM) of the methyltransferase inhibitor, 5‐azacytidine (Emani *et al*., 2002). Treated seedlings had significantly shorter roots than controls (Fig. 4d,e), similar to observations in Arabidopsis (Virdi *et al*., 2015). To investigate if DNA methylation also plays a role in root tissue specification, we generated whole‐genome bisulfite sequencing data for identical tissue types to those profiled for RNA‐seq experiments (Fig. 1a). Whole‐root and whole‐shoot organs and vascular and nonvascular tissues were collected from the root meristem and elongation zones. DNA methylome data were generated by subjecting DNA to sodium bisulfite conversion before DNA sequencing for each sample in three biological replicates. Biological replicates were highly correlated (Table S9) and were combined to obtain average genome coverage of 5–12× (Tables S10–S13), similar coverage to that previously described in rice and maize (Gent *et al*., 2013; Stroud *et al*., 2013). The average genome‐wide methylation levels (weighted methylation levels) (Schultz *et al*., 2012) was extremely similar between samples in the CHH, CG and CHG contexts (Fig. 4c; Widman *et al*., 2014).

Our methylation data were analyzed to determine whether these four methylomes, including those generated from LCM coupled with bisulfite sequencing, were comparable to typical whole‐genome bisulfite sequencing data. In all instances, CG and CHG methylation was prevalent close to the centromere, while CHH methylation was distributed more evenly across the chromosomes (Figs 4a, S6) as described for a number of methylomes in several species (Cokus *et al*., 2008; Li *et al*., 2012; Regulski *et al*., 2013; Zhong *et al*., 2013b). Next we determined whether the broad methylation patterns were largely similar across genic regions (Fig. 4b). CG methylation was the most predominant of the three contexts and was highest in the exons and dipped immediately 3′ and 5′ of genes. CHG methylation was lowest in exons as previously described but also demonstrated a peak not previously described at the 5′ end of the coding sequence. CHH methylation was lower than the other two contexts, although small increases within coding regions were observed, relative to the 5′ and 3′ regions, which has not been observed in rice, Arabidopsis or maize (Figs 4b, S6). With the exception of the 5′ CHG coding region peak and the slight increase in CHH coding region methylation, these patterns were consistent across all tissues and organs (Fig. S6) and are the same as reports of plant methylomes in Arabidopsis, maize, rice, sorghum and tomato (Zhang *et al*., 2006, 2011; Lister *et al*., 2008; Wang *et al*., 2009; Li *et al*., 2012; Zhong *et al*., 2013b; Olson *et al*., 2014).

**Figure 5:**
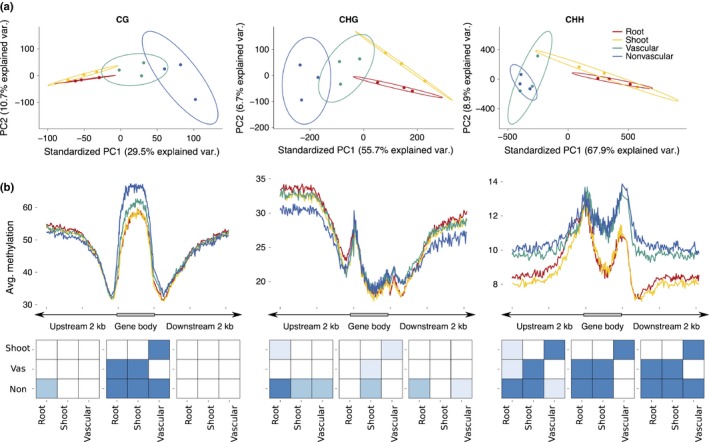
Distribution of methylation across genomic features. (a) Principal component (PC) analysis on methylation state (methylated or unmethylated) for all potential cytosines of each methylation context shared between the four tissue types (root, shoot, vascular and nonvascular) for three biological replicates. (b) Methylation density in each context, CG, CHG and CHH, averaged in all genes with mapping coverage of ≥ 4× in root, shoot, vascular and nonvascular samples. The bottom panel denotes significant differences in average methylation of the genomic region (upstream, gene body, downstream) between samples (root, shoot, vascular, nonvascular), where the darkest blue represents *P*‐values ≤ 0.0001, the second darkest are *P*‐values ranging from 0.0001 to 0.001, and the lightest blue are *P*‐values in the range 0.001–0.01 determined by a Tukey test.

Having established that our data followed previously described patterns (Figs 4a,b, S6), we carried out direct comparisons to determine if tissue‐specific differences in DNA methylation exist between samples. Discrimination of variation in the methylation status for individual cytosines (methylated or unmethylated) was observed using PCA (Fig. 5a). Our inclusion of biological replicates confirmed replicate reliability and demonstrated that differences in CG, CHG and CHH methylation can be used to distinguish vascular and nonvascular tissues as well as root and shoot organs. In the CG and CHG context, vascular and nonvascular tissues separated in the first principal component and root and shoot organs separated across the second (Fig. 5a). In the CHH context, root and shoot organs separated from vascular nonvascular tissues across the first principal component, yet there was little separation within tissue or organ types across components (Fig. 5a). The separation of tissues observed in each context demonstrates that variance in cytosine methylation state is sufficient to distinguish tissue and organ types. Furthermore, increased variation in tissues vs organs suggests that tissue‐specific bisulfite sequencing data capture data at a higher resolution than obtained using whole organs.

We next set out to determine where this variation arises with respect to upstream regulatory and downstream regulatory regions and the gene body. Average methylation levels in each context are similar between tissues and organs (Fig. 4c); however, their patterns differ significantly in specific genic regions (Fig. 5b). CG methylation was significantly elevated (*P *<* *0.0001) in the gene body of vascular and nonvascular tissues relative to the shoot and root (Fig. 5b). Significant differences in CHG methylation levels were observed in the 5′ upstream regulatory region between root and nonvascular samples (*P *<* *0.0001), root and shoot organs (*P *=* *0.006), and vascular and nonvascular samples (*P *=* *0.0001). Consistent with the PCA, significant differences in CHH methylation were predominantly observed between whole organs and tissues in all regions of the gene body, where organs were methylated at a much lower level than that observed for nonvascular and vascular tissues (Fig. 5b, *P* < 0.0001). Thus, methylation in distinct contexts differs in a tissue‐ and organ‐specific manner dependent on gene features, context and tissue type.

### Methylation in genic regions varies with tissue type and expression level

To determine whether the methylation changes observed between the four samples were correlated with changes in gene expression, we cross‐referenced our methylome and RNA‐seq transcriptome datasets (Figs S7, S8). Genes expressed in all samples were placed in groups based upon their expression level quartile in that tissue/organ (referred to as groups 1–4, with group 1 demonstrating lowest expression and group 4 the highest). Methylation levels were compared for each context within each tissue/organ with respect to each expression group (Fig. S7). To determine if differences in methylation levels between varying levels of RPKM were significant, a nonparametric ANOVA followed by a Tukey test was run on each genic region (2 kb upstream of the gene, 2 kb downstream and within the gene body). In CG and CHG contexts, average methylation differed significantly (*P *<* *0.0001) with varying levels of expression within all samples (Fig. S7). In the CG context, average methylation differed predominantly within the gene body of genes (*P *<* *0.0001) for each tissue/organ, and in the CHG context average methylation differed with gene expression (*P *<* *0.0001) in regions 5′ upstream of coding regions in all tissues/organs and within the gene body in roots, shoots and vascular tissue. In the CHH context, average methylation differed significantly (*P *<* *0.0001) only within root and shoot organs in the regions within the gene body and regions 3′ of the stop codon (Fig. S7). Thus, methylation patterns within and surrounding the gene body are associated with changes in gene expression.

**Figure 6:**
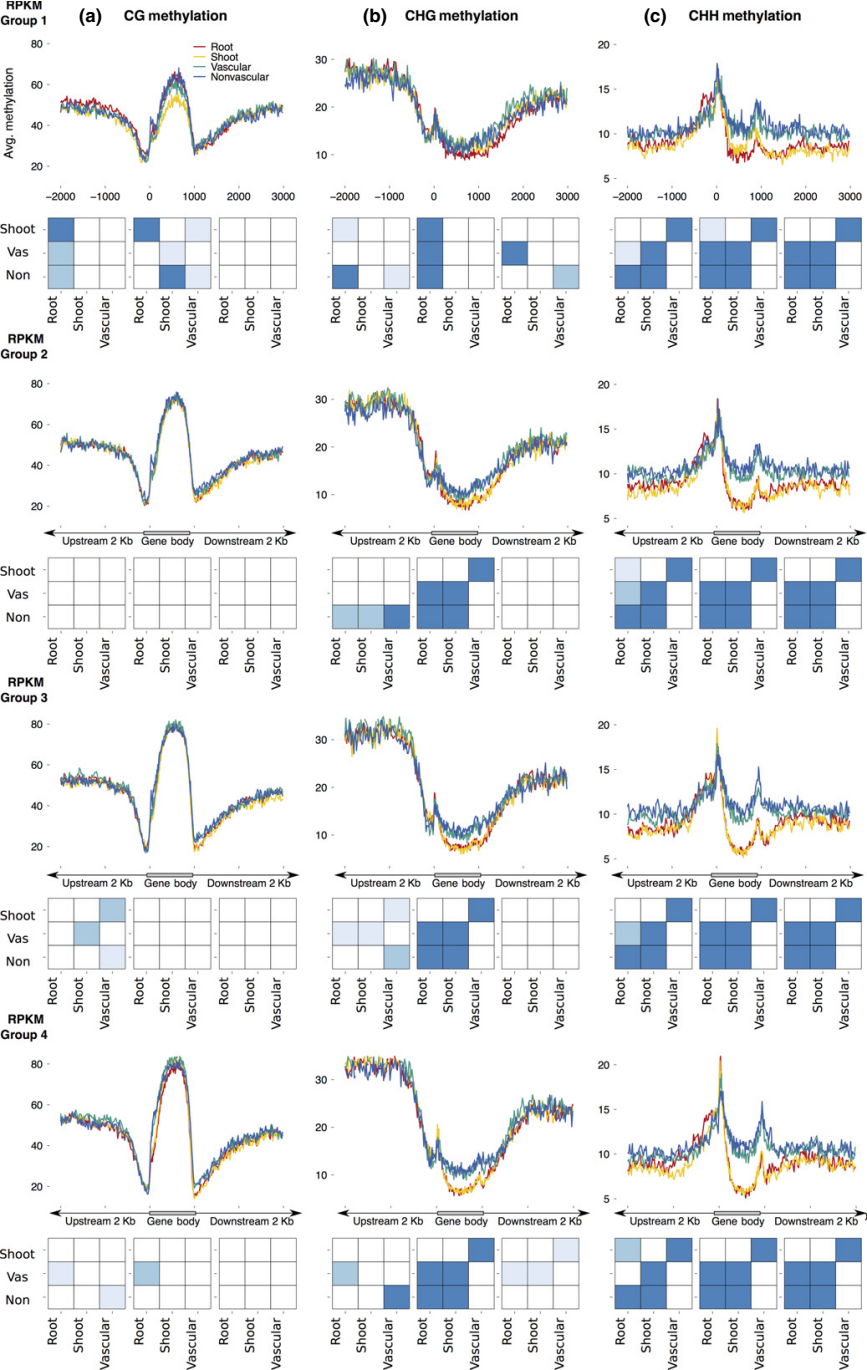
Methylation in genic regions varies with tissue type and tissue‐specific expression level. Average methylation levels of genic regions for each expression level group from each sample. Genes were categorized into four groups based on their quartile of expression level in each sample, where group 1 is the quartile containing genes of lowest expression level and group 4 is the quartile containing genes with the highest expression level. The methylation level of each group in each sample is shown for each context; CG (a), CHG (b) and CHH (c). The bottom panel denotes significant differences in average methylation of the genomic region (upstream, gene body, downstream) between samples (root, shoot, vascular, nonvascular), where the darkest blue represents *P*‐values ≤ 0.0001, the second darkest are *P*‐values ranging from 0.0001 to 0.001, and the lightest blue are *P*‐values in the range 0.001–0.01 determined by a Tukey test.

To further determine if methylation changes were associated with changes in gene expression in a tissue‐specific manner, we explored differences between tissue and organ type for each expression quartile. For CG methylation (Fig. 6a), lowly expressed genes varied between the tissue/organ types in their methylation levels within exons (*P *<* *0.0001), but, by contrast, differences were nearly indistinguishable in higher expression quartiles. The most prominent change was observed in shoot tissue, which had low methylation in the gene body compared with the root and vascular/nonvascular tissue of lowly expressed genes. Methylation in the CHG context (Fig. 6b) also varied depending on expression levels; however, in contrast with that observed in the CG context, differences were observed predominantly in expression quartiles 2, 3 and 4. In this context, tissue from the shoot and root demonstrated lower methylation than that of vascular and nonvascular root tissues in exonic regions (*P *<* *0.0001) (Fig. 6b, lower panels). CHH methylation was considerably higher in root vascular and nonvascular tissues in comparison to that of the root and shoot (Fig. 6c). These differences were apparent in all four expression quartiles and became greater with increasing levels of expression (*P *<* *0.0001). Thousands of genes contribute to these changes in methylation between samples as expression increases, suggesting that changes in methylation are at a global level between tissues (Table S14).

**Figure 7:**
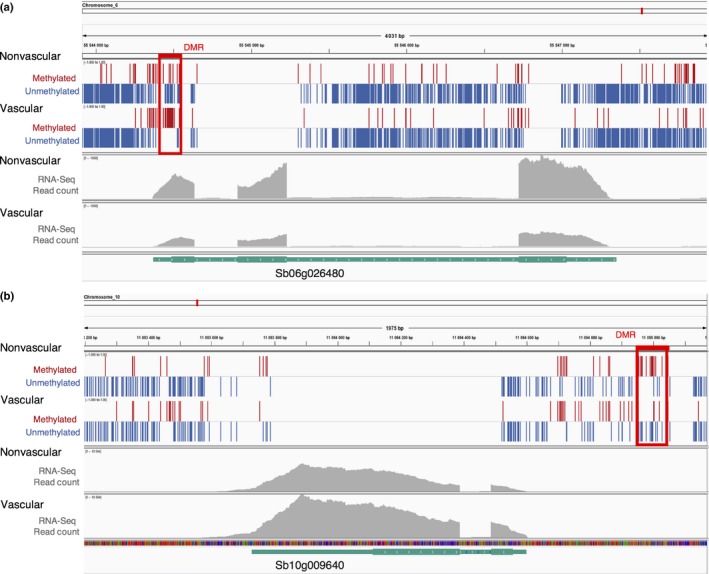
Differentially methylated regions between vascular and nonvascular tissues. Selected differentially methylated regions (DMRs) that overlap with differentially expressed genes. All three methylation contexts were merged to identify DMRs. The first few panels colored cytosines as either red (methylated) or blue (unmethylated) depending on their methylation status. Regions boxed in red represent DMRs found between vascular and nonvascular samples. The following panels show the number of RNA‐Seq reads (in gray) mapping to each gene in vascular and nonvascular tissues. The gene model and the direction of the gene are indicated by the turquoise track and white arrowheads. Differences in read count between vascular and nonvascular tissues are differentially expressed following edgeR analysis. Gene annotations are shown below in green, and exons are represented by larger squares. Both the expansin precursor *Sb06g026480* and gibberellin‐regulated gene *Sb10g009640* are differentially expressed.

In order to determine whether differential methylation might be associated with genes regulating vascular development in the root, we identified differentially methylated regions (DMRs) between vascular and nonvascular tissue (Table S15). Regions with a *P*‐value ≤ 0.001 and at least 70% methylation in one tissue and < 30% in the other tissue were identified as DMRs (similar to Regulski *et al*., 2013). In all, 1576 DMRs were identified between vascular and nonvascular tissue types. Of these, 621 were associated with genic regions (Table S15); 156 root vascular/nonvascular differentially expressed genes were associated with root vascular/nonvascular DMRs. A subset of these genes have previously been shown to have roles in Arabidopsis root development (Tables 2, S15). Cell elongation in the root is controlled by GA, particularly in the endodermis (Ubeda‐Tomas *et al*., 2008; Ubeda‐Tomás *et al*., 2009), and the gene encoding a putative gibberellin receptor, *Sb03g005570*, and gibberellin‐responsive genes *Sb06g019290* and *Sb10g009640* (Tables 2, S15) demonstrated differential methylation in addition to differential expression. The cell elongation process is closely associated with remodelling of the primary cell wall (Dolan & Davies, 2004) and it is of note that the genes for a cellulose synthase enzyme *CESA2* (*Sb01g004210*) and an expansin (*Sb06g026480*; Fig. 7) were both differentially methylated and expressed between vascular and nonvascular tissues.

**Table 2:** Genes with differential expression associated with differentially methylated regions

Gene ID	Description	Increased expression	Increased methylation
Sb06g026480	Expansin	Nonvascular	Vascular
Sb01g027880	CSLD1 – cellulose synthase‐like family D	Nonvascular	Vascular
Sb03g005570	Gibberellin receptor GID1L2	Nonvascular	Vascular
Sb06g019290	OsGASR4 – gibberellin‐regulated	Vascular	Vascular
Sb10g024830	Strubbelig receptor family 7	Vascular	Vascular
Sb04g012910	Nodulin MtN3 family protein	Vascular	Vascular
Sb10g021750	BES1/BZR1 homolog protein	Vascular	Vascular
Sb03g037510	OsFBX27 – F‐box domain	Vascular	Vascular
Sb10g009640	OsGASR7 – gibberellin‐regulated	Vascular	Nonvascular
Sb10g028580	Glycosyl hydrolases family 16	Vascular	Nonvascular
Sb07g020920	Linker histone H1 and H5 family	Vascular	Nonvascular
Sb01g004210	CESA2 – cellulose synthase	Vascular	Nonvascular

To identify conserved hypermethylated genomic regions within vascular tissue, we compared our sorghum vascular genic hypermethylated regions (Table S16) with an Arabidopsis stele‐specific set (Kawakatsu *et al*., 2016). The CG context has the highest percentage of conserved hypermethylated regions (69%) (Fig. S9), while CHG and CHH both have much lower conservation (16% and 14%, respectively). Conserved CG genes also showed enrichment for GO terms related to both vascular development and DNA methylation (Table S16). Some of these GO terms include the glucuronoxylan metabolic process (*P *=* *9.37e–05), regulation of DNA endoreduplication (*P *=* *0.002 09), root meristem growth, gravitropism (*P *=* *1.44e–08), polysaccharide biosynthetic process (*P *=* *0.002 47), chromatin remodeling (*P *=* *0.035) and production of siRNA involved in RNA interference (*P *=* *1.44e–08). In all, 70% of these conserved vascular CG hypermethylated genes belong to higher vascular expression level quartiles 3 and 4 (chi‐squared with Yate correction, *P *>* *0.0001). These data support a role for DNA methylation in vascular development, and specifically CG methylation where high gene‐body methylation often results in higher gene expression.

## Discussion

We have explored genes linked with vascularization in sorghum using tissue‐specific transcriptome profiling and coexpression analyses, and identified both known and uncharacterized genes with enriched expression in the developing root vasculature (Figs 1, 3; Tables [Link], [Link]). We observed a considerable expansion of several transcription factor families when we compared our sorghum vascular‐expressed genes with those of Arabidopsis and maize. NAC, WRKY, MYB, and ABI transcription factor families were overrepresented in root vascular tissue of sorghum relative to Arabidopsis and maize. Other families, including ALF and ARF, were expanded in maize and sorghum compared with Arabidopsis, and HB, bZIP, HSF and GRAS transcription factors demonstrated enrichment in vascular tissue of maize and Arabidopsis relative to sorghum. Expansion of these families suggests differential wiring of the underlying regulatory networks in each species for vascular development. Two overrepresented transcription factor families in sorghum, NACs and MYBs are widely associated with xylem maturation in dicots (Kubo *et al*., 2005; Zhong *et al*., 2006, 2008, 2011, 2013a; Mitsuda *et al*., 2007; Zhou *et al*., 2009; Zhong & Ye, 2012). SCWs of monocots demonstrate greater lignin content than that present in their dicot counterparts, and one possibility is that these differences represent different SCW specializations. It will be interesting to see if vascular‐expressed transcription factor families that have undergone expansion in sorghum (Fig. 2) are, in part, responsible for such monocot‐specific differences.

Our identification of an enrichment of transcripts associated with methylation and epigenetic regulation was surprising. An investigation of the Arabidopsis root vascular transcriptome revealed similar functional enrichments, suggesting that our observations in sorghum may be common to higher plants (Fig. 2b). We demonstrated that methylation differs across the body of genes that are highly expressed in the vascular tissue. A handful of studies have characterized DNA methylation in distinct tissues, organs and over developmental time. Recently, profiled cell types of the Arabidopsis root show CHH hypermethylation of transposable elements in the columella, potentially owing to a loss of heterochromatin and a corresponding increase in small RNA expression (Kawakatsu *et al*., 2016). Here, vascular tissue did covary relative to other tissues, as observed with our data in sorghum for CG and CHH methylation. Additional studies profiling the tomato leaf and fruit revealed differences in CG body methylation levels (Zhong *et al*., 2013b). The Arabidopsis embryo has considerably higher levels of CG methylation relative to the endosperm, which undergoes active DNA demethylation (Hsieh *et al*., 2009). Levels of maintenance DNA methyltransferases are higher in the dividing tissue of maize leaves than that present in mature tissue, with a number of CCGG sequences showing differential methylation at the transition from cell division to leaf expansion (Candaele *et al*., 2014). Together, these studies demonstrate that differences in gene‐body methylation and enzymes associated with DNA methylation can be observed based on tissue or developmental stage. Here, we have observed differential expression of genes involved in DNA methylation between root vascular and nonvascular tissue and corresponding differences in levels of CG, CHG and CHH methylation between sorghum vascular and nonvascular tissues, as well as in the root and the shoot. Furthermore, variation in methylation of each type can distinguish tissue and organ types using PCA (Fig. 5). Like cell type or tissue‐resolution expression profiling, tissue‐specific bisulfite sequencing data capture at a higher resolution than that obtained using whole organs. Indeed, from the PCA, for CG and CHG methylation, the majority of the variation observed could be attributed to differences between tissues.

Functional consequences of CG gene‐body methylation are controversial. CG gene‐body methylation was first reported in Arabidopsis and found to be associated with highly expressed genes (Zhang *et al*., 2006). Body‐methylated genes tend to be longer and were described as ‘more functionally important’ than unmethylated genes, on the basis that mutations were more likely to result in phenotypes than lesions in genes not demonstrating high levels of gene‐body methylation. Genes reported to demonstrate high levels of body methylation were also reported to evolve more slowly than unmethylated genes (Takuno & Gaut, 2012). Loss of CG methylation in the *met1* mutant background does not substantially affect the expression of body‐methylated genes (Zhang *et al*., 2006) and consequently a number of hypotheses with respect to the functional significance of gene‐body methylation have arisen. These include suppression of expression from cryptic promoters within coding regions, accurate splicing, and/or methylation as a byproduct of transcription (Lorincz *et al*., 2004; Zilberman *et al*., 2007; Roudier *et al*., 2009; Teixeira & Colot, 2009; Luco *et al*., 2010; Maunakea *et al*., 2010, 2013). Our data show a surprising relationship with respect to levels of CG‐body methylation and gene expression. Differences in gene‐body methylation were prevalent in lowly expressed genes between tissues and organs (the first quartile of expression levels; Fig. 6a). Developmental identity may play a role in determining these differences. Within the gene body, higher levels of vascular and nonvascular CHG and CHH methylation relative to the root and shoot were also observed. These differences were dramatic in higher expression level quartiles. CHG methylation is thought to hinder transcriptional elongation (Miura *et al*., 2009); however, our observation does not support this hypothesis given that genes in all quartiles have roughly similar profiles. To the best of our knowledge, the observed changes in CHH methylation in the gene body between tissues and organs had never been reported before (Figs 5a, 6c). Our observation that these levels are further magnified with increasing expression level suggests that developmental identity and transcription may be interlinked in the CHH context.

At the gene level, a number of studies point to dynamic control of DNA methylation very early in development via imprinting. In particular, differential methylation of parental alleles of *FERTILIZATION INDEPENDENT ENDOSPERM 2* were shown to occur in the maize endosperm (Gutierrez‐Marcos *et al*., 2006). Studies have also suggested that methylation changes of individual genes may be a feature of proliferating tissue. The Arabidopsis gene *PHABULOSA*, which has a critical role in patterning in several developmental contexts (McConnell *et al*., 2001; Emery *et al*., 2003; Prigge *et al*., 2005), including the root xylem (Carlsbecker *et al*., 2010), is hypomethylated in meristematic tissues (Bao *et al*., 2004). We have identified DMRs in genes that are associated with regulation of the phytohormone GA, and in genes that mediate the GA response (Fig. 7; Table 2). GA has been widely reported as being required for cell elongation (Ogas *et al*., 1997; Collett *et al*., 2000; Ubeda‐Tomas *et al*., 2008), including in vascular tissue of Arabidopsis (Ragni *et al*., 2011) and hybrid aspen, where increases in GA also lead to increased radial growth – a hallmark of vascular proliferation (Eriksson *et al*., 2000). In root development, interplay between the auxin and gibberellin determine exit from the root meristem and entry to the elongation zone (Moubayidin *et al*., 2010). Here, GA‐dependent cell elongation occurs before cellular differentiation.

In conclusion, our study has identified changes in gene expression between sorghum vascular root tissue and nonvascular root tissue, whole roots and whole shoots. In addition to expected overrepresentation of biological processes associated with vascularization in plants, in root vascular mRNA profiles, we also observed enrichment in the complement of specific transcription factor families. These results suggest that differences in the regulatory logic determine differences in vascular cell specification, differentiation and/or function in sorghum relative to maize and Arabidopsis. Our further identification of genes associated with DNA methylation as being differentially regulated in vascular tissue was supported by the identification of differentially methylated genomic regions in this tissue, thus providing functional analysis in support of our transcriptomic data. A number of DMRs are associated with genes influencing growth. Furthermore, we have identified differences in overall levels of CG gene‐body methylation that have been previously shown to differ in tomato tissues. However, we show that these differences appear magnified in the more lowly expressed genes, which has not been previously demonstrated. Differences in gene‐body CHG and CHH methylation also vary by tissue and increase in magnitude when considering more highly expressed genes.

## Author contributions

S.M.B., D.W., C‐L.W., J.P.E., J.S., G.M.T., P.K. and K.K. planned and designed the research. S.D., C‐L.W., K.K., J.P.E., G.M.T., C‐Y.N. and B.W. conducted experiments. G.M.T., G.K‐R., A.O., D.T., S.K. and S.S. preformed data analysis. J.P.E., G.M.T. and S.M.B. wrote the original draft. All authors helped with editing.

## Supporting information

Please note: Wiley Blackwell are not responsible for the content or functionality of any Supporting Information supplied by the authors. Any queries (other than missing material) should be directed to the *New Phytologist* Central Office.

**Fig. S1** High correlation of FPKM values among biological replicates from whole root and shoot.
**Fig. S2** High correlation of FPKM values among biological replicates from root vascular and nonvascular tissues.
**Fig. S3** Comparison of qRT‐PCR and RNA‐seq data for LCM collected samples.
**Fig. S4** Abundance of transcription factor families across species.
**Fig. S5** Overlap in vascular coexpressed genes.
**Fig. S6** Sorghum epi‐genome has a similar distribution across samples.
**Fig. S7** Distribution of methylation marks across genes of varying expression levels.
**Fig. S8** Distribution of cytosines across genes of varying expression levels.
**Fig. S9** Orthologous vascular hypermethylation.

**Table S1** Sorghum qRT‐PCR primers used for testing tissue‐specific expression

**Table S2** RNA‐seq data read counts and mapping efficiency from shoot and root sorghum tissues
**Table S3** RNA‐seq data read counts and mapping efficiency from vascular and nonvascular tissues in sorghum

**Table S4** Sorghum root and shoot differential expression

**Table S5** Sorghum vascular and nonvascular differential expression

**Table S6** GO terms for vascular‐expressed genes from all tissues for sorghum and vascular‐expressed genes in maize and Arabidopsis

**Table S7** Vascular‐expressed genes that are orthologous in maize, sorghum and Arabidopsis

**Table S8** Sorghum vascular‐expressed gene coexpression with *VND7*,* CESA4*, and *Bmr2* and their corresponding GO categories of enrichment

**Table S9** Pearson correlation ran on methylation status of each cytosines
**Table S10** Bisulfite‐seq data read count and mapping and coverage statistics for vascular and nonvascular tissues in sorghum
**Table S11** Bisulfite‐seq read count, mapping and coverage statistics of combined replicates for vascular, nonvascular, whole root and shoot of sorghum
**Table S12** Bisulfite‐seq data read count and mapping and coverage statistics for sorghum whole‐root and ‐shoot samples
**Table S13** CG, CHG and CHH Bisulfite‐seq coverage statistics for sorghum whole‐root, whole‐shoot samples and vascular and nonvascular laser‐dissected samples

**Table S14** Number of sorghum genes contributing to average methylation changes between tissues and reads per kilobase per million (RPKM) groups

**Table S15** Sites of sorghum vascular and nonvascular differentially methylated regions along with the genes with which these differentially methylated regions may be associated

**Table S16** Vascular hypermethylated regions conserved between sorghum and Arabidopsis vascular‐specific sodium bisulfite sequencing data for the CG, CHG and CHH context along with their corresponding GO terms
