# Supplementary material for: DNA methylation and gene expression regulation associated with vascularization in Sorghum bicolor
Source: New Phytol. 2017 Feb 10;214(3):1213–29. doi: 10.1111/nph.14448 (PMC5655736; doi:10.1111/nph.14448)

New Phytologist

***New Phytologist* Supporting Information**

Article title: **DNA methylation and gene expression regulation associated with vascularization in *Sorghum bicolor***

Authors: Gina M. Turco, Kaisa Kajala, Govindarajan Kunde-Ramamoorthy, Chew-Yee Ngan, Andrew Olson, Shweta Deshphande, Denis Tolkunov, Barbara Waring, Scott Stelpflug, Patricia Klein, Jeremy Schmutz, Shawn Kaeppler, Doreen Ware, Chia-Lin Wei, J. Peter Etchells and Siobhan M. Brady[1+]

Article acceptance date: 19 December 2016

The following Supporting Information is available for this article:

Figs S1–S9 in this file; Tables S1–S16, see separate files.

Fig. S1 High correlation of FPKM values among biological replicates from whole root and shoot. Shoot biological replicates (SXHZ, TOGA, TOGB) compared to root biological replicates (TOGC, TOGG, TOGH) in Pearson correlation.

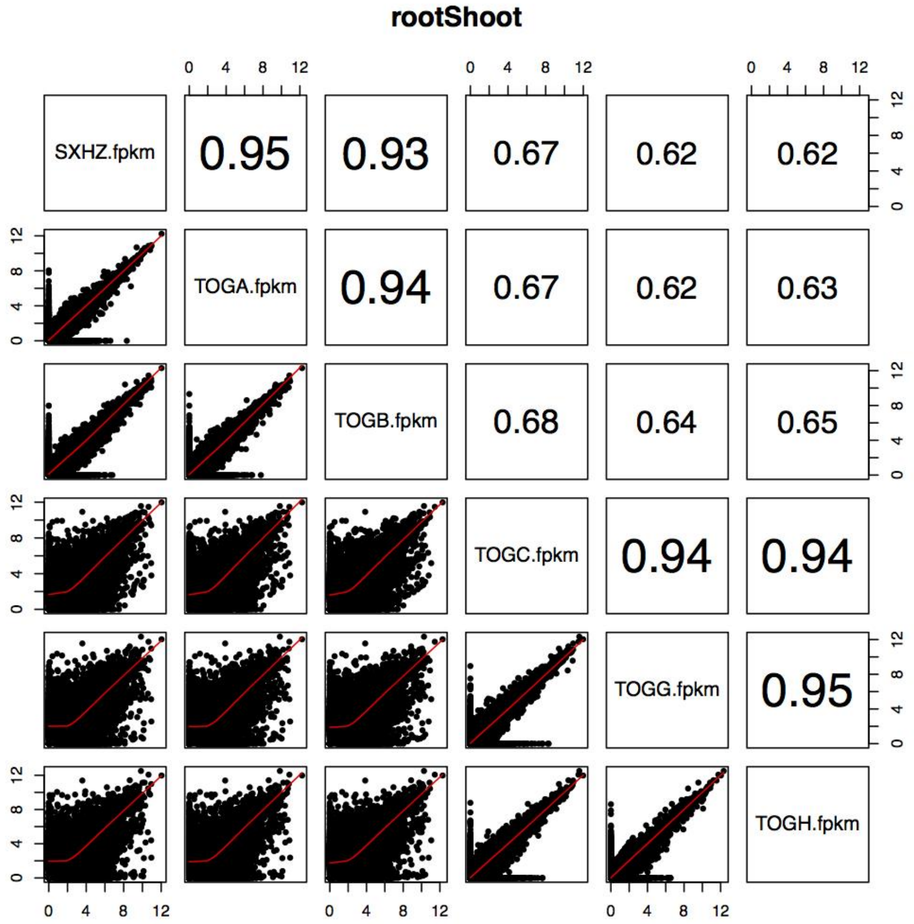

Fig. S2 High correlation of FPKM values among biological replicates from root vascular and nonvascular tissues. Vascular biological replicates (NABO,NABN,NABS) compared to nonvascular biological replicates (NABP,NABT,NABU) in Pearson correlation.

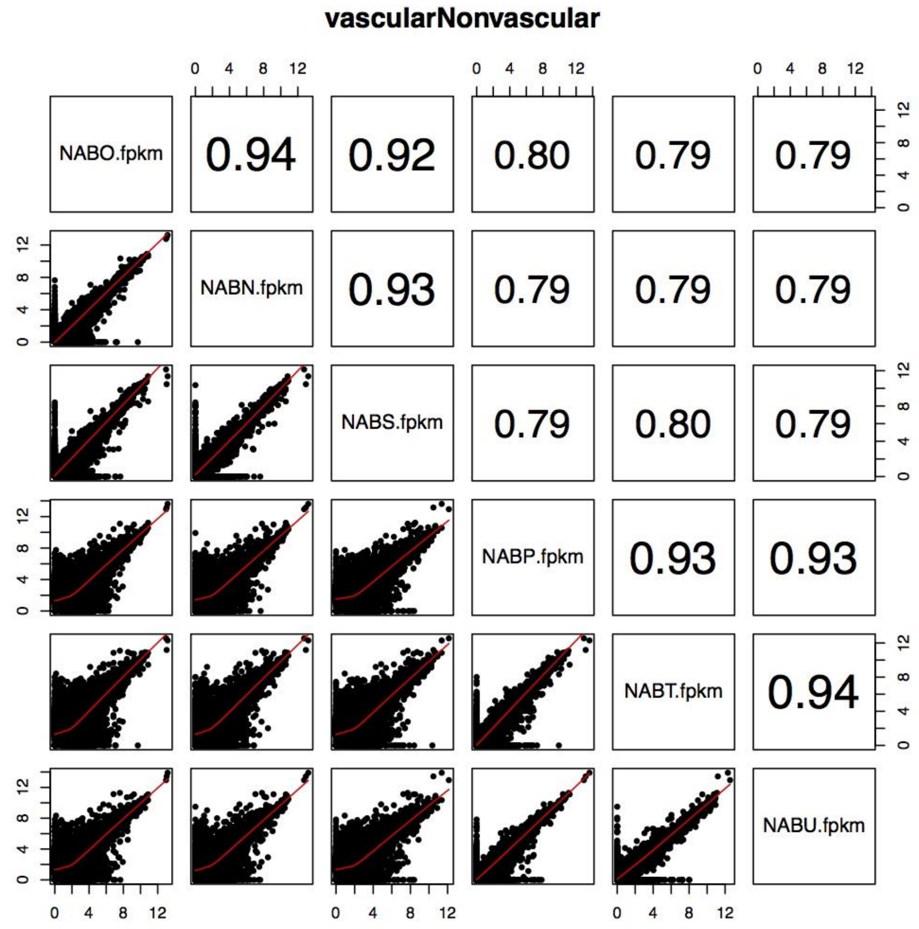

Fig. S3 Comparison of qRT-PCR to RNA-seq data for LCM collected samples. RNA-seq and qRT-PCR generate similar profiles of transcript abundance from LCM material. Ratios of relative transcript abundance in vascular and non-vascular tissues were calculated. Blue bars represent the ratios determined from two biological replicates of qRT-PCR and the red bars represent the ratios determined from normalized read counts of three biological replicates of RNA-seq. The error bars represent standard error between biological replicates.

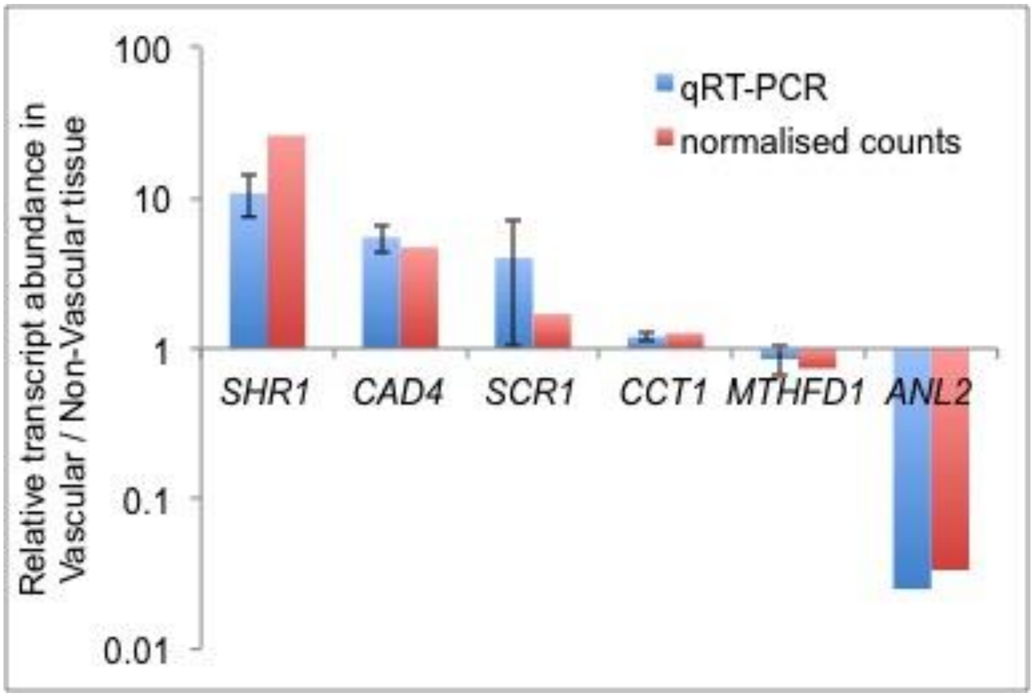

Fig. S4 Abundance of transcription factor families across species. **(A)** ARF transcription factor family phylogeny across sorghum, maize and Arabidopsis, with branch lengths included. Filled ticks represent vascular-enriched gene within the ARF family. Sorghum and maize have an expansion of ARFs in comparison to Arabidopsis. **(B)** Number of transcription factors from each family in sorghum , maize , Arabidopsis. Only transcription factors found in all three species are shown. The shading of red is dependent on the number of transcription factors in that family, where a darker red indicated more transcription factors.

New Phytologist

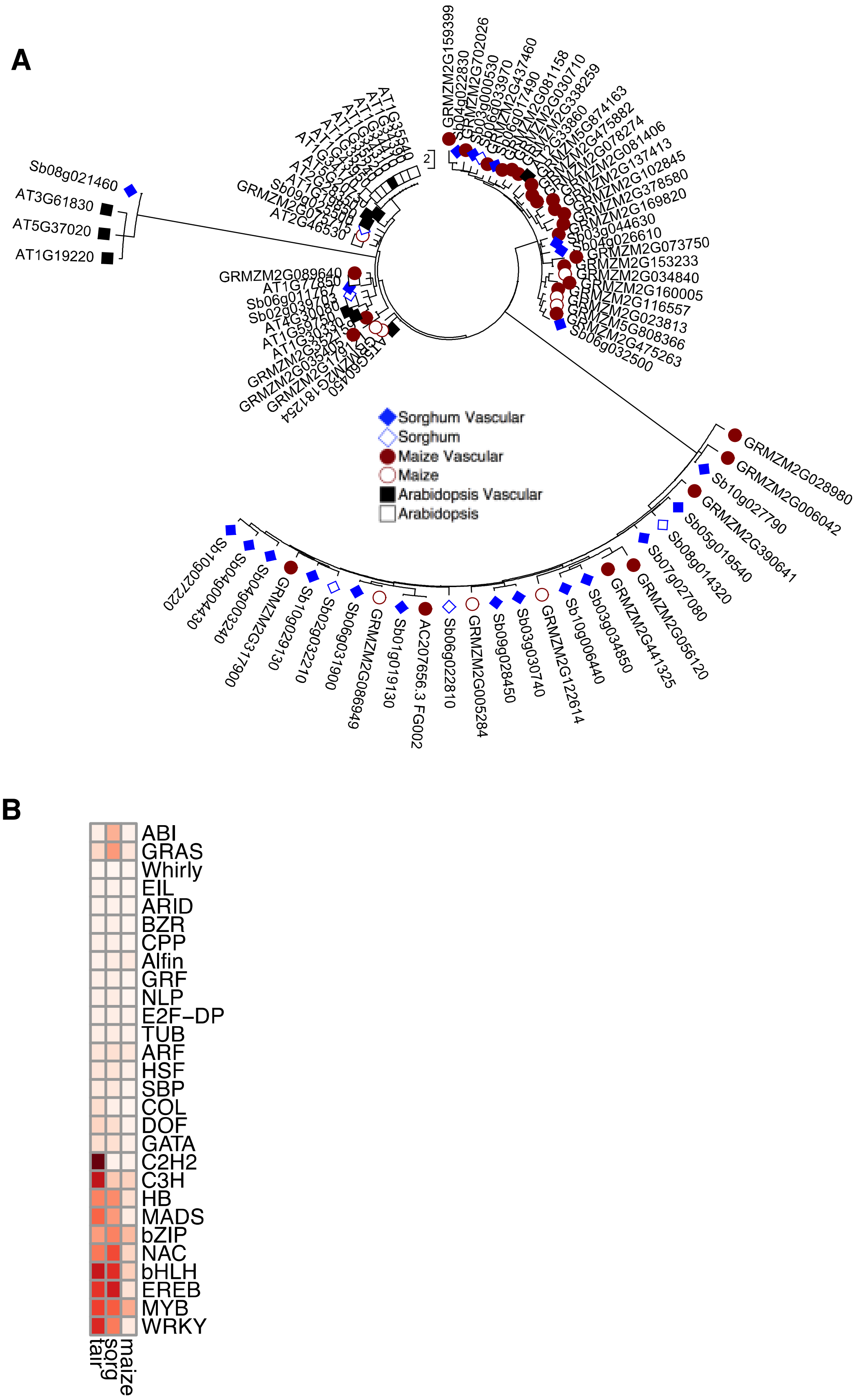

Fig. S5 Fig.

Fig. S5 Overlap in vascular co-expressed genes. Number of genes co-expressed with *Bmr2* **(A)**, *VND7* **(B)** or *CESA4* **(C)** with a correlation higher than 0.9 and their overlap with vascular-enriched genes. **(D** and **E)** The amount of overlap between *Bmr2*, *VND7* and *CESA4* co-expressed with a correlation > 0.9 for all genes and then filtered for vascular-enriched only genes, respectively.

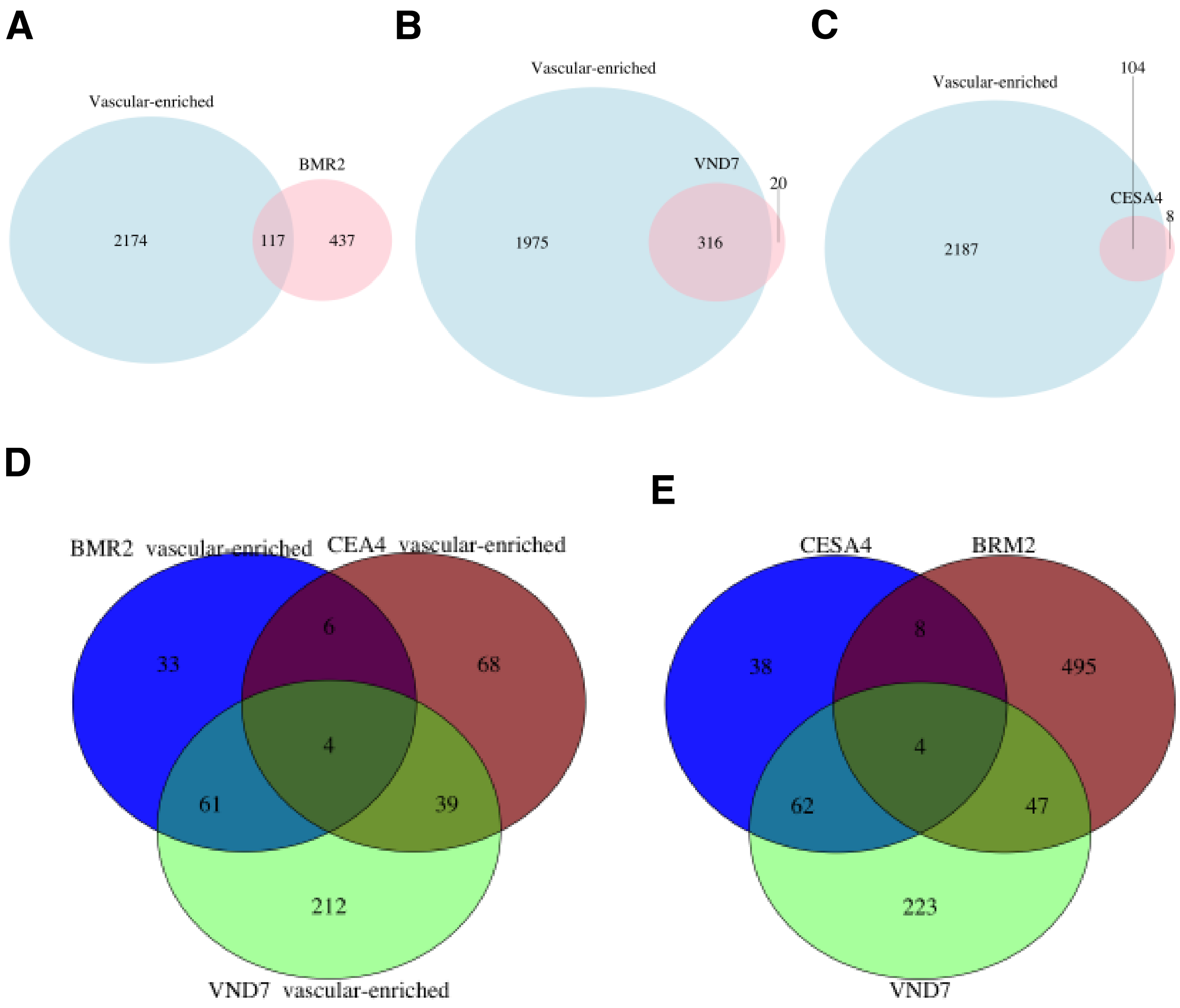

Fig. S6 Sorghum epi-genome has a similar distribution across samples. **(A)** distribution of methylation marks in all three contexts for all four samples. Increased methylation is indicated by a darker blue for the region. Areas with no coverage are white. **(B)** Distribution of methylation marks averaged across the gene-body of all genes with >= 4X coverage for each sample.

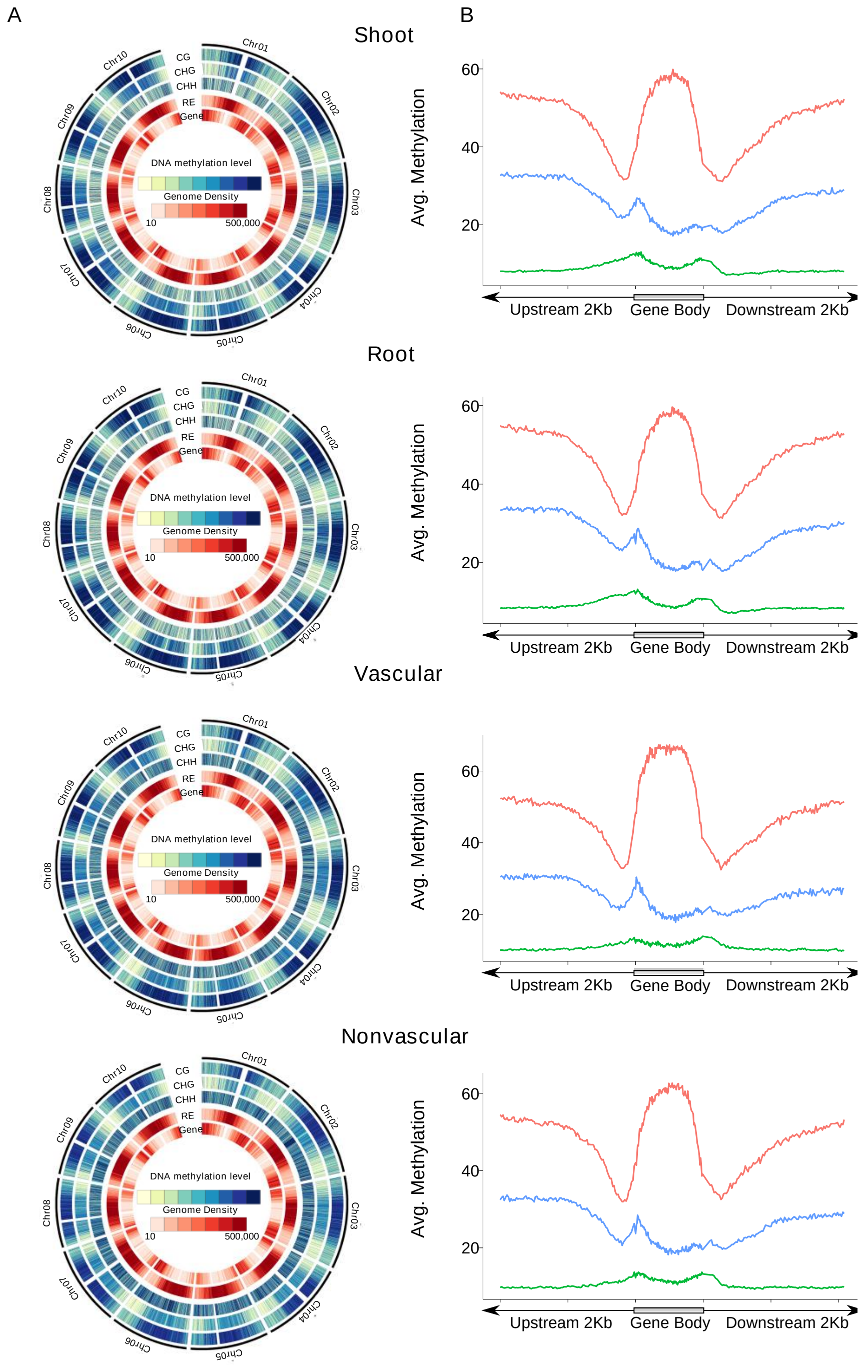
A
B
Shoot
Root
Vascular
Nonvascular
CG
CHG
CHH
RE
Gene
DNA methylation level
Genome Density
10
500,000
Chr01
Chr02
Chr03
Chr04
Chr05
Chr06
Chr07
Chr08
Chr09
Chr10
Avg. Methylation
60
40
20
Upstream 2Kb
Gene Body
Downstream 2Kb

Fig. S7 Distribution of methylation marks across genes of varying expression levels. Distribution of methylation marks averaged across the gene-body of all genes with >= 4X coverage for each sample in each methylation context. Genes were categorized into four groups based on their quartile of expression in each tissue type. Group 1 delineates the lowly expressed genes and group 4 the highest expression quartile. Each group is represented by different shade of red, green or blue depending on the methylation context. The bottom panel denotes significant differences in average methylation of the genomic region (upstream, gene body, downstream) between reads per kilobase per million (RPKM) groups 1-4. Where the darkest blue represents *P*-values less than or equal to 0.0001 the second darkest are *P*-values ranging from 0.0001-0.001 and the lightest blue are *P*-values of 0.001-0.01 determined by a Tukey test.

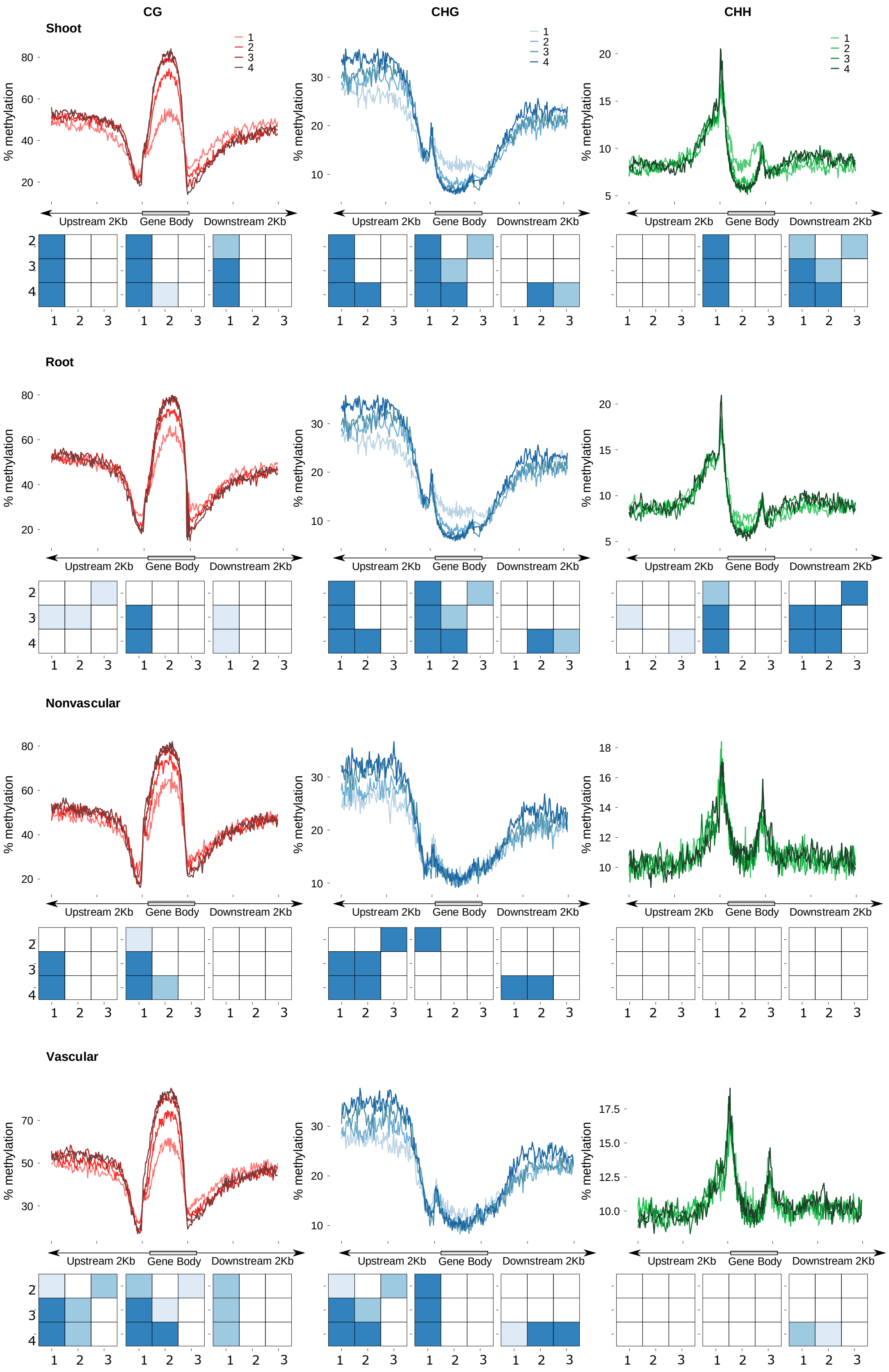
CG
CHG
CHH
Shoot
Root
Nonvascular
Vascular
% methylation
Upstream 2Kb
Gene Body
Downstream 2Kb
1
2
3
4

Fig. S8 Distribution of cytosine's across genes of varying expression levels. Distribution of potential sites for each methylation context averaged across the gene-body of genes for each binned expression quartile. Group 1 delineates the lowly expressed genes and group 4 the highest expression quartile. Each group is represented by different shade of red, green or blue depending on the methylation context.

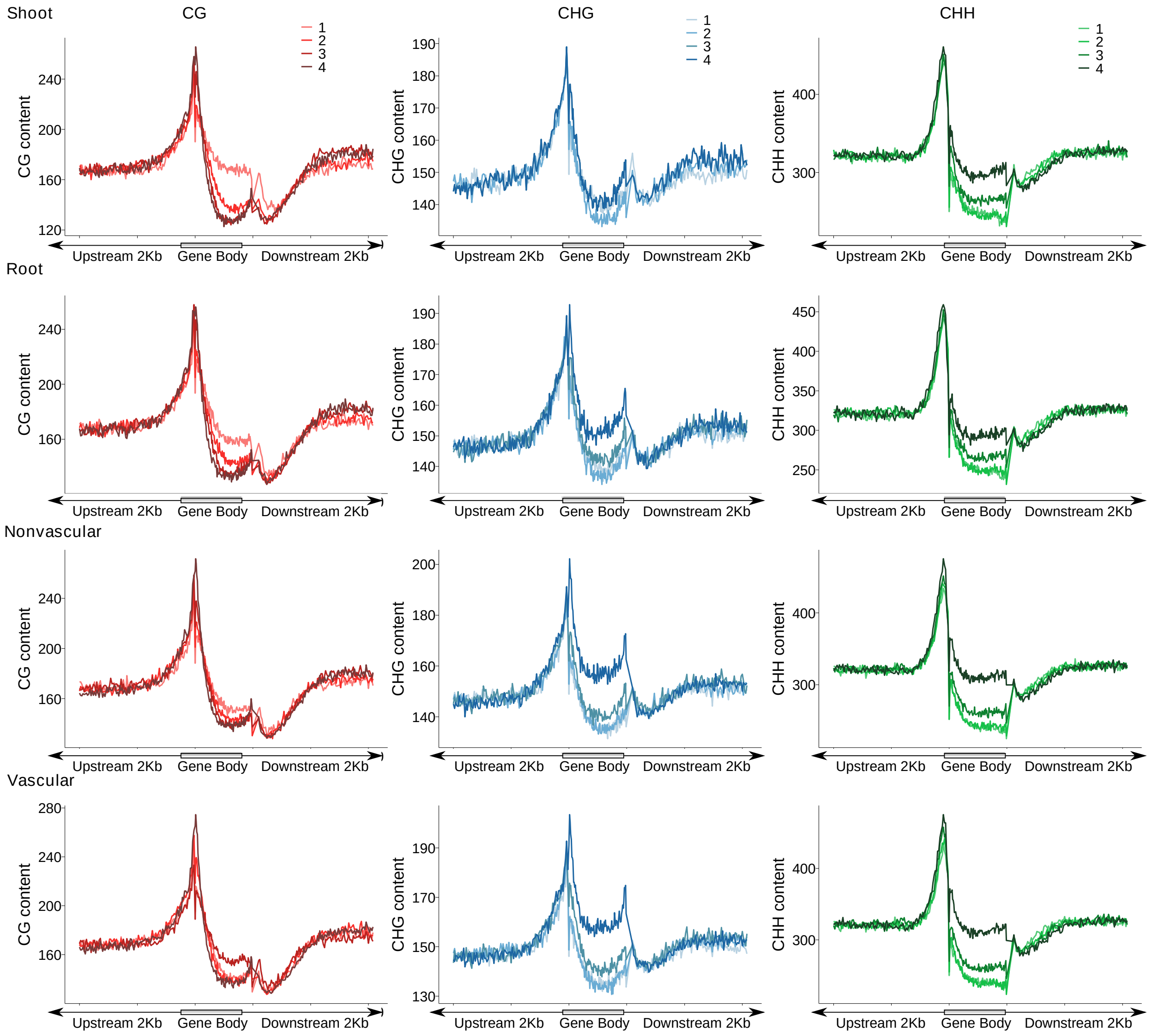

Fig. S9 Orthologous vascular hyper-methylation.Orthologous gens between Arabidopsis and sorghum that have been classified as hyper-methylated among vascular specific Sodium Bisulfite Sequencing data for each methylation context; CG **(A)**, CHG **(B)** and CHH **(C)**. Genes are classified using a one tailed binomial test on regions with more than 20 cytosine's and at least 4X coverage. Genes with significantly higher methylation than the genome average were considered hyper-methylated.

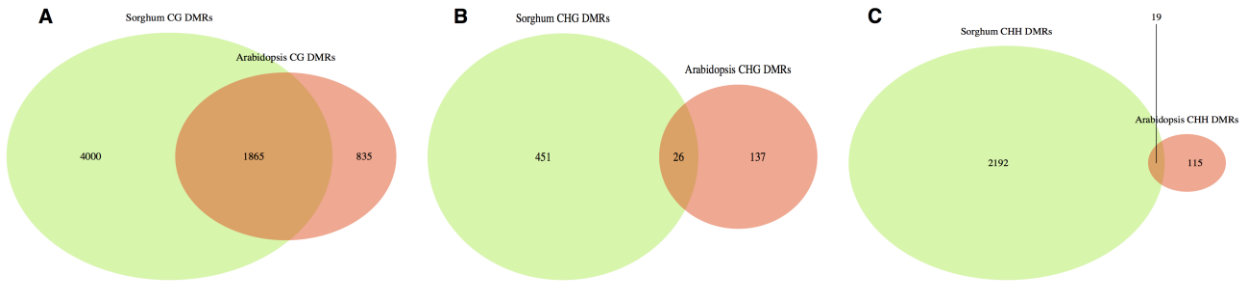

Supplement: Supplementary file 1 — Fig. S1 High correlation of FPKM values among biological replicates from whole root and shoot. Fig. S2 High correlation of FPKM values among biological replicates from root vascular and nonvascular tissues. Fig. S3 Comparison of qRT‐PCR and RNA‐seq data for LCM collected samples. Fig. S4 Abundance of transcription factor families across species. Fig. S5 Overlap in vascular coexpressed genes. Fig. S6 Sorghum epi‐genome has a similar distribution across samples. Fig. S7 Distribution of methylation marks across genes of varying expression levels. Fig. S8 Distribution of cytosines across genes of varying expression levels. Fig. S9 Orthologous vascular hypermethylation. [file NPH-214-1213-s001.pdf]
