# Supplementary material for: DNA methylation and gene expression regulation associated with vascularization in Sorghum bicolor
Source: New Phytol. 2017 Feb 10;214(3):1213–29. doi: 10.1111/nph.14448 (PMC5655736; doi:10.1111/nph.14448)
Supplement: Supplementary file 3 — Table S2 RNA‐seq data read counts and mapping efficiency from shoot and root sorghum tissues Table S3 RNA‐seq data read counts and mapping efficiency from vascular and nonvascular tissues in sorghum [file NPH-214-1213-s003.pdf]

Library name	Sample name	Raw reads	Filtered reads	% Transcriptome mapping	Genome coverage
SXHZ	Sorghum bicolor Shoot RNA -1	86139964	81086734	78.6	13
TOGA	Sorghum bicolor Shoot RNA -2	103395862	98025420	79.0	16
TOGB	Sorghum bicolor Shoot RNA -3	115003066	108465156	80.5	18
TOGC	Sorghum bicolor Root RNA -1	117405732	111392450	82.1	19
TOGG	Sorghum bicolor Root RNA -2	80950200	76939870	82.2	13
TOGH	Sorghum bicolor Root RNA -3	115202118	109821062	82.2	18.6

Supporting Information Table S2 RNA-seq data read counts and mapping efficiency from shoot and root sorghum tissues. High percentages (80%) of the reads are mapped to the reference transcriptome.

Library name	Sample name	Raw	Filtered reads	% Transcriptome mapping	Genome coverage
NABO	Vascular A	69057188	57623998	70.9	8.4
NABN	Vascular B	70328874	62329636	73.0	9.4
NABS	Vascular C	68802918	60954674	76.5	9.6
NABP	Non-vascular A	76890240	67534120	71.3	9.9
NABT	Non-vascular B	63750594	58835700	77.0	9.4
NABU	Non-vascular C	74019060	65094296	71.5	9.6

Supporting Information Table S3 RNA-seq data read counts and mapping efficiency from vascular and nonvascular tissues in sorghum. More than 70% of the reads are aligned to the reference transcriptome.
