# Supplementary material for: DNA methylation and gene expression regulation associated with vascularization in Sorghum bicolor
Source: New Phytol. 2017 Feb 10;214(3):1213–29. doi: 10.1111/nph.14448 (PMC5655736; doi:10.1111/nph.14448)
Supplement: Supplementary file 9 — Table S9 Pearson correlation ran on methylation status of each cytosines Table S10 Bisulfite‐seq data read count and mapping and coverage statistics for vascular and nonvascular tissues in sorghum Table S11 Bisulfite‐seq read count, mapping and coverage statistics of combined replicates for vascular, nonvascular, whole root and shoot of sorghum Table S12 Bisulfite‐seq data read count and mapping and coverage statistics for sorghum whole‐root and ‐shoot samples Table S13 CG, CHG and CHH Bisulfite‐seq coverage statistics for sorghum whole‐root, whole‐shoot samples and vascular and nonvascular laser‐dissected samples [file NPH-214-1213-s009.pdf]

Tissue	Biological Replicate 1	Biological Replicate 2	Biological Replicate 3
Vascular 1 CG	1	1	1
Vascular 2 CG	1	1	0.99
Vascular 3 CG	1	0.99	1
Nonvascular 1 CG	1	0.99	0.99
Nonvascular 2 CG	0.99	1	0.99
Nonvascular 3 CG	0.99	0.99	1
Root 1 CG	1	1	1
Root 2 CG	1	1	1
Root 3 CG	1	1	1
Shoot 1 CG	1	1	1
Shoot 2 CG	1	1	1
Shoot 3 CG	1	1	1

Tissue	Biological Replicate 1	Biological Replicate 2	Biological Replicate 3
Vascular 1 CHG	1	0.99	0.99
Vascular 2 CHG	0.99	1	0.99
Vascular 3 CHG	0.99	0.99	1
Nonvascular 1 CHG	1	0.99	0.99
Nonvascular 2 CHG	0.99	1	0.99
Nonvascular 3 CHG	0.99	0.99	1
Root 1 CHG	1	1	1
Root 2 CHG	1	1	1
Root 3 CHH	1	1	1
Shoot 1 CHG	1	1	1
Shoot 2 CHG	1	1	1
Shoot 3 CHG	1	1	1

Tissue	Biological Replicate 1	Biological Replicate 2	Biological Replicate 3
Vascular 1 CHH	1	0.96	1
Vascular 2 CHH	0.96	1	0.97
Vascular 3 CHH	0.96	0.97	1
Nonvascular 1 CHH	1	0.97	0.97

Nonvascular 2 CHH	0.97	1	0.97
Nonvascular 3 CHH	0.97	0.97	1
Root 1 CHH	1	0.97	0.98
Root 2 CHH	0.97	1	0.98
Root 3 CHH	0.98	0.98	1
Shoot 1 CHH	1	0.98	0.98
Shoot 2 CHH	0.98	1	0.98
Shoot 3 CHH	0.98	0.98	1

Supporting Information Table S9 Pearson correlation ran on methylation status of each cytosines. Comparing biological replicates with atleast 4X coverage in across all replicates in all samples; sorghum whole root, shoot, vascular and nonvascular.

**Tissue**	**Vascular**			**Non-vascular**		
Library Name	N0030	N0033	N0036	N0029	N0032	N0035
Raw Reads	218870346	105307118	89411338	194153264	127264072	106251360
Filtered Reads	181420256	83245874	72857584	157743786	103542154	85965044
Uniquely mapped reads (PE)	67,385,007 (74 %)	28,529,686 (69 %)	25,082,087 (69, %)	56,975,357 (72 %)	37,221,701 (72 %)	25,567,372 (60 %)
Non-redundant reads	19,296,520 (29 %)	16,200,106 (57 %)	10,556,267 (42 %)	9,183,455 (17 %)	14,203,892 (38 %)	10,798,294 (42 %)
# Cytosines covered (>=1)	207,751,782 (68 %)	235,028,538 (77 %)	167,376,769 (55 %)	167,013,863 (54 %)	200,732,147 (66 %)	188173554 (61 %)
# Cytosines covered (>=4)	76,113,499 (25 %)	65,601,660 (33 %)	31,611,936 (17 %)	25,979,980 (8.4 %)	54,771,994 (18 %)	30,451,993 (13 %)

Supporting Information Table S10 Bisulfite-seq data read count and mapping and coverage statistics for vascular and nonvascular tissues in sorghum. Around 70 % of the reads are mapped to reference genome. Only 10 – 25 % of the Cytosines show read depth of at least 4.

Tissue	Root			Shoot		
Library Name	WPOB	WPNZ	WPOA	WPOC	WPOG	WPOH
Raw Reads	192,934,242	164,895,258	130,980,690	148,217,754	189,949,424	166,650,304
Filtered Reads	168,301,762	143,950,384	114,284,736	130,111,590	163,924,852	145,185,994
Uniquely mapped reads (PE)	63,478,450 (75 %)	39,191,973 (54 %)	46,229,953 (81 %)	42,704,200 (66 %)	53,918,330 (66 %)	44,942,635 (62 %)
# cytosines covered (>=1)	260,237,172 (84.9%)	224,197,516 (73.2%)	249,161,375 (81.3%)	230,632,759 (75.3%)	257,655,947 (84%)	223,951,011 (73%)
# cytosines covered (>=3)	180,293,049 (58.8%)	110,384,456 (36%)	162,743,821 (53.1%)	129,746,019 (42.3%)	171,855,432 (56.1%)	110,008,906 (35.9%)
# cytosines covered (>=4)	146,505,227 (47.8%)	77,359,441 (25.2%)	130,409,914 (42.5%)	97,741,938 (31.9%)	136,656,841 (44.6%)	76,618,241 (25%)

Supporting Information Table S11 Bisulfite-seq read count, mapping and coverage statistics of combined replicates for vascular, nonvascular, whole root and shoot of sorghum.

Tissue	Root	Shoot	Vascular	Nonvascular
Genome Coverage	11X	12X	6X	5X
# cytosines covered (>=1)	300,445,151 (98%)	299,548,221 (97.8%)	283,514,796 (92.5%)	265,099,838 (86.5%)
# cytosines covered (>=3)	252,246,678 (82.3 %)	246,490,588 (80.4 %)	207,444,704 (67.7%)	175,772,390 (57.3%)
# cytosines covered (>=4)	228,152,012 (74.5 %)	221,239,937 (72.2 %)	173,966,757 (56.8%)	142,086,083 (46.4%)

Supporting Information Table S12 Bisulfite-seq data read count and mapping and coverage statistics for sorghum whole root and shoot samples. Around 60 % of the reads are mapped to reference genome.

Tissue CG	Vascular	Non-vascular	Root	Shoot
Min	0	0	0	0
1st Quartile Range	1	0	2	2
Mean	4.280785669403951	3.367311585228629	8.35860559961259	7.64619262611591
Median	3	2	5	4
3rd Quartile Range	6	4	10	9
Max	7694	6379	14847	22275

Tissue CHG	Vascular	Non-vascular	Root	Shoot
Min	0	0	0	0
2nd Quartile Range	1	1	2	2
Mean	4.954484714131013	3.927147486767571	9.54176952723960	8.75820393683714
Median	3	2	6	5
3rd Quartile Range	7	5	12	11
Max	7450	6141	13922	21194

Tissue CHH	Vascular	Non-vascular	Root	Shoot
Min	0	0	0	0
2nd Quartile Range	2	1	3	3
Mean	5.893018880232094	4.72797129700005	11.2572552160533	10.5566784161498
Median	4	3	7	7
3rd Quartile Range	8	6	14	13
Max	7720	6357	15270	22724

Supporting Information Table S13 CG, CHG and CHH Bisulfite-seq coverage statistics for sorghum whole root, whole shoot samples and vascular and nonvascular laser dissected samples.
